# Evaluation of extraction methods for untargeted metabolomic studies for future applications in zebrafish larvae infection models

**DOI:** 10.1038/s41598-023-34593-y

**Published:** 2023-05-09

**Authors:** Philip Schippers, Sari Rasheed, Yu Mi Park, Timo Risch, Lea Wagmann, Selina Hemmer, Sascha K. Manier, Rolf Müller, Jennifer Herrmann, Markus R. Meyer

**Affiliations:** 1 Department of Experimental and Clinical Toxicology, Center for Molecular Signaling (PZMS), Institute of Experimental and Clinical Pharmacology and Toxicology, Saarland University, 66421 Homburg, Germany; 2 Helmholtz Institute for Pharmaceutical Research Saarland (HIPS), Helmholtz Centre for Infection Research (HZI), Saarland University, Saarbrücken, Germany; 3 German Centre for Infection Research (DZIF), Partner Site Hannover, Braunschweig, Germany

**Keywords:** Mass spectrometry, Drug development, Bioanalytical chemistry

## Abstract

Sample preparation in untargeted metabolomics should allow reproducible extractions of as many molecules as possible. Thus, optimizing sample preparation is crucial. This study compared six different extraction procedures to find the most suitable for extracting zebrafish larvae in the context of an infection model. Two one-phase extractions employing methanol (I) and a single miscible phase of methanol/acetonitrile/water (II) and two two-phase methods using phase separation between chloroform and methanol/water combinations (III and IV) were tested. Additional bead homogenization was used for methods III and IV (III_B and IV_B). Nine internal standards and 59 molecules of interest (MoInt) related to mycobacterial infection were used for method evaluation. Two-phase methods (III and IV) led to a lower feature count, higher peak areas of MoInt, especially amino acids, and higher coefficients of variation in comparison to one-phase extractions. Adding bead homogenization increased feature count, peak areas, and CVs. Extraction I showed higher peak areas and lower CVs than extraction II, thus being the most suited one-phase method. Extraction III and IV showed similar results, with III being easier to execute and less prone to imprecisions. Thus, for future applications in zebrafish larvae metabolomics and infection models, extractions I and III might be chosen.

## Introduction

Metabolomics aims to analytically profile changes of molecules < 1500 Da (metabolome) present in an organism or a model system at a certain time point^1,2^. The metabolome includes endogenous metabolites as well as metabolites originating from exogenous sources such as drugs. While targeted approaches are based on the quantification of selected metabolites, often from a particular group of metabolites, untargeted metabolomics aims to detect as many metabolites as possible^1–3^. Often applied analytical techniques for the sample separation include liquid chromatography (LC) and gas chromatography, which both are regularly coupled with mass spectrometry^1–3^. LC separation in untargeted approaches is often done using reversed-phase columns for the separation of non-polar metabolites, normal-phase columns for the separation of polar metabolites or hydrophilic interaction liquid chromatography (HILIC) columns as variations of normal-phase columns, employing a water layer on top of their stationary phase, resulting in a separation based mostly on liquid–liquid partitioning. Phenyl-Hexyl columns are reversed-phase columns with unique selectivity for aromatic molecules. After separation of the metabolites, they are ionized, commonly using electrospray ionization (ESI) or atmospheric pressure ionization and analyzed using a mass analyzer, often Orbitrap or time-of-flight instruments^1,2^. Fragmentation spectra can be generated in the same analysis using for example data-dependent or data-independent acquisition, or in a subsequent analysis. The so generated data can then be further processed using bioinformatics methods such as peak detection and preprocessing, followed by multivariate statistics for evaluation and comparison of the fragmentation spectra against reference libraries for identification^1–5^.

To understand the effect of diseases, for example tuberculosis^6^, and to find biomarker for a diagnosis or treatment success, metabolomics is often applied and the change of the host metabolome is observed. Specifically, changes to the endogenous metabolome originating from *Mycobacterium tuberculosis* infection were extensively studied in humans^4–7^ and animal models^8^ including mice, guinea pigs, rabbits, and non-human primates^9^. The induced changes on the metabolome were also studied using the non-tuberculous mycobacterium *M. marinum* in zebrafish larvae (*Danio rerio*, ZF) as one of its natural hosts^10^. Such ZF model using *M. marinum* resembles the granuloma like structures, typically found in its mammalian counterpart^11–14^. The ZF genome is about 70% identical to that of humans^15^ and in several studies a similar metabolism to humans, both regarding xenobiotic metabolism^16–19^, as well as host metabolome in case of infection^20,21^ was reported. The ZF larvae model has further advantages, which include the ease of handling, optical transparency of embryos and larvae, and lower monetary costs, compared to other organisms^22,23^. Furthermore, experiments performed with embryo and larvae that are younger than 120 h post-fertilization (hpf) are not considered as animal experiments within the European Union (EU directive, 2010/63/EU)^24^. Due to the multitude of advantages, ZF studies employing untargeted metabolomics are nowadays more and more common^25,26^, but rarely used in the context of diseases and *M. marinum* in particular^20^.

During preliminary studies conducted for the implementation of a *M. marinum* infection model in ZF larvae, the lack of sample material due to the small size of ZF larvae amongst led to low signal intensity of metabolites and resulted in missed metabolites and poor repeatability. Optimization of the employed methods, especially the sample preparation was deemed necessary. To cover a sufficiently large portion of the metabolome, combinations of different methods for extraction, separation, or identification are often used^1–3,27,28^.

Commonly used liquid extractions for ZF larvae and other fish tissue are often based on one-phase extractions, which consist of one or more miscible solvents, such as methanol, acetonitrile, and water^29^ or solely methanol^19^. They are also based on two-phase methods, consisting of two immiscible solutions^30^, utilizing phase separation between a lipophilic and hydrophilic phase. The most common two-phase methods use one polar solution, e.g., mixtures of methanol and water and one apolar solution, e.g., chloroform^31,32^, methyl tert-butyl ether^33^ or heptane^34^.

Another approach is the application of additional sample homogenization. While some tissue samples can be extracted directly using solvents, more resistant tissues need additional break down steps. Those methods include chemical homogenization, which have the disadvantage of possible interference with the metabolome or the following extraction processes, or mechanical homogenization of the larvae by e.g., grinding, beating the larvae with beads or ultra-sonification^35^.

This study aimed to compare different sample extraction procedures, including one-phase, two-phase, and two-phase with mechanical homogenization, to find the most suitable one for the future use in a *M. marinum* ZF larvae infection model. Different methods were investigated based on multiple quality assessment strategies, involving internal standards, so-called “Molecules of Interest” (MoInt), and pitfalls as well as improvements elucidated.

## Methods

Pronase and methylene blue were obtained from Sigma-Aldrich (Taufkirchen, Germany). NaCl, KCl, MgSO_4_, Ca(NO_3_)_2_, and 4-(2-hydroxyethyl)-1-piperazineethanesulfonic acid (HEPES) were obtained from Carl Roth (Karlsruhe, Germany). Tryptophan-d_5_ was obtained from Alsachim (Illkirch Graffenstaden, France). Telmisartan-d_7_ was purchased from Sigma (Taufkirchen, Germany). Trimipramin-d_3_, bisoprolol-d_5_, furosemide, glibenclamid and hydrochlorothiazide were obtained from LGC (Wesel, Germany). Ammonium formate, ammonium acetate (both analytical grade), formic acid (LC–MS grade), d-glucose-d_7_ and palmitic acid-d_31_ were purchased from Merck (Darmstadt, Germany). Acetonitrile (ACN, LC–MS grade), methanol (MeOH, LC–MS grade), and all other chemicals and reagents (analytical grade) were from VWR (Darmstadt, Germany). Chloroform (analytical reagent grade) was from Fisher (Schwerte, Germany). Deionized water was produced by a Millipore system (18.2 Ω × cm water resistance) from Merck (Darmstadt, Germany). Reaction tubes and pipette tips were obtained from Sarstedt (Nümbrecht, Germany). 2 mL homogenizer tubes prefilled with acid washed 0.5 mm glass beads were obtained from Biozym (Hess, Germany).

### Sample preparation

Husbandry of adult ZF was carried out in accordance with the German Animal Welfare Act (§11 Abs. 1 TierSchG) and based on previously documented methods^19,36^. ZF larvae within the first 120 hpf are not considered as animal experiments according to the EU Directive 2010/63/EU. The zebrafish maintenance, embryo preparation and the sample collection up to the sample preparation can be found in the supplementary material.

Two one-phase extractions (I–II) and two two-phase extractions (III–IV) were investigated, with extraction I used for preliminary experiments. The two-phase extractions were additionally evaluated in combination with mechanical pre-treatment, using glass bead homogenization.

For bead homogenization, indicated as “_B” in the results, 0.5 mm glass beads from prefilled homogenization tubes (Biozym, Hess, Germany) were added to the shock frozen samples. The samples were homogenized using a MP Biomedicals FastPrep24 homogenizer (TF, Dreieich, Germany) for 20 s at 6 m/s in the Quick Prep mode and kept on ice afterwards. Three different spike solutions (1, 2 and 3), containing different IS were used during the extraction. Their content and the peak picking of the IS, as well as *m/z* and retention time can be found in Table S1.

For extraction I, adapted from Park et al., 180 µL of MeOH and 20 µL internal standard solution spike solution 1 (25 µg/mL tryptophan-d_5_, 1 mg/mL palmitic acid-d_31_, 50 µg/mL glucose-d_7_ in MeOH) were added to each sample^19^. The samples were vortexed for 10 s and afterwards centrifuged at 4 °C and 21,500×*g* for 10 min.

For extraction II, adapted from Bai et al., 80 µL of MeOH, 100 µL of ACN and 40 µL of Millipore water, together with 20 µL spike solution 1 were added to each sample^29^. The samples were sonicated using an USC 100T ultrasonic cleaner (VWR, Darmstadt, Germany) for 15 min at room temperature, incubated at − 20 °C for 2 h and afterwards centrifuged at 4 °C and 21,500×*g* for 15 min.

For extractions III and III_B, adapted from Chai et al., 180 µL of MeOH and 80 µL of Millipore water, together with 20 µL spike solution 1 were added to each sample^37^. 200 µL chloroform and 100 µL Millipore water were added and the samples were vortexed for 1 min. The samples were incubated on ice for 10 min and then centrifuged at 4 °C and 13,800×*g* for 10 min.

**Figure 1:**
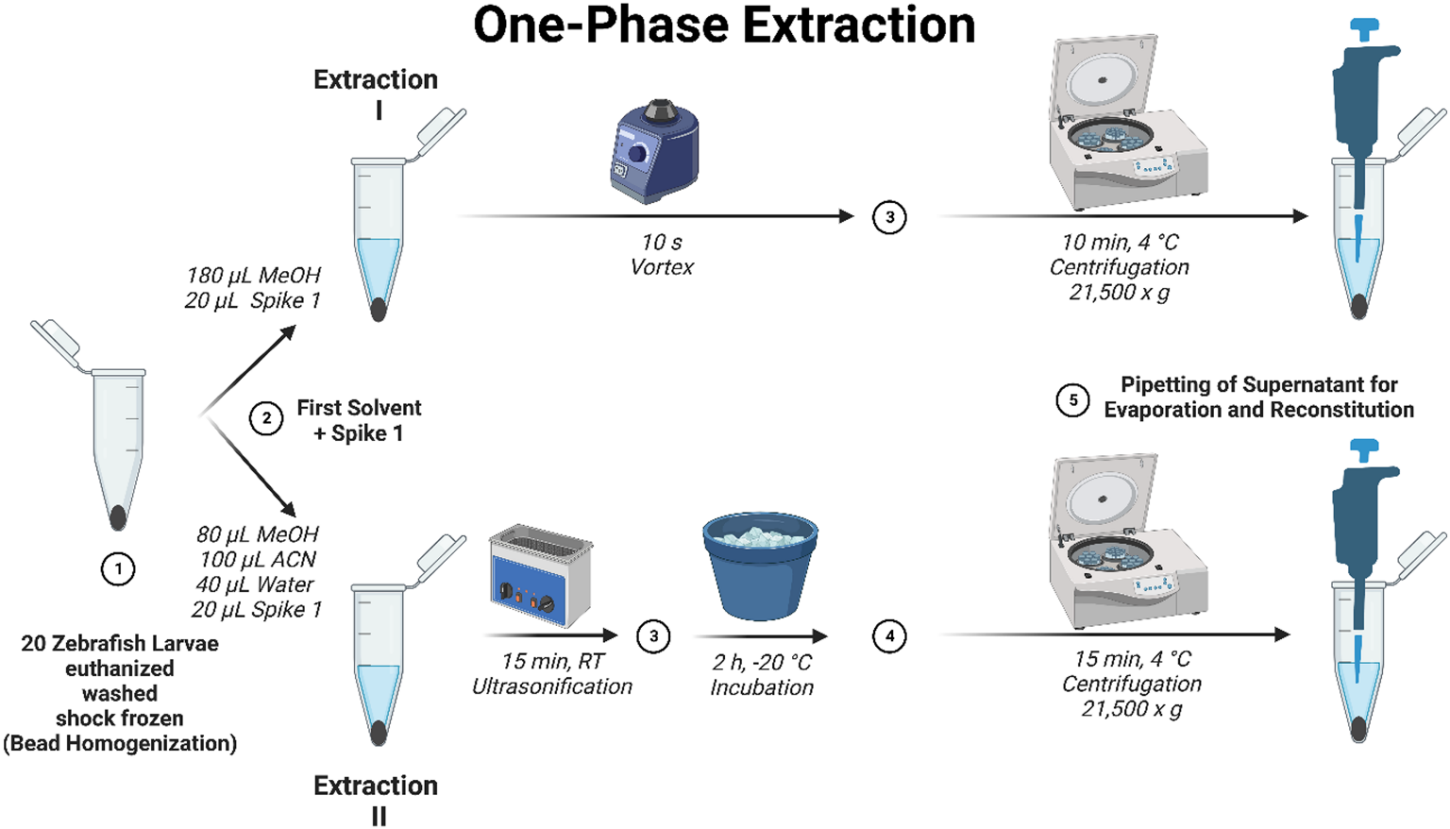
Workflow for extraction procedures I and II. *MeOH* Methanol, *ACN* Acetonitrile. *Spike 1 solution* 25 µg/mL tryptophan-d_5_, 1 mg/mL palmitic acid-d_31_, 50 µg/mL glucose-d_7_ in MeOH. Generated by BioRender.com and published based on their academic license terms.

**Figure 2:**
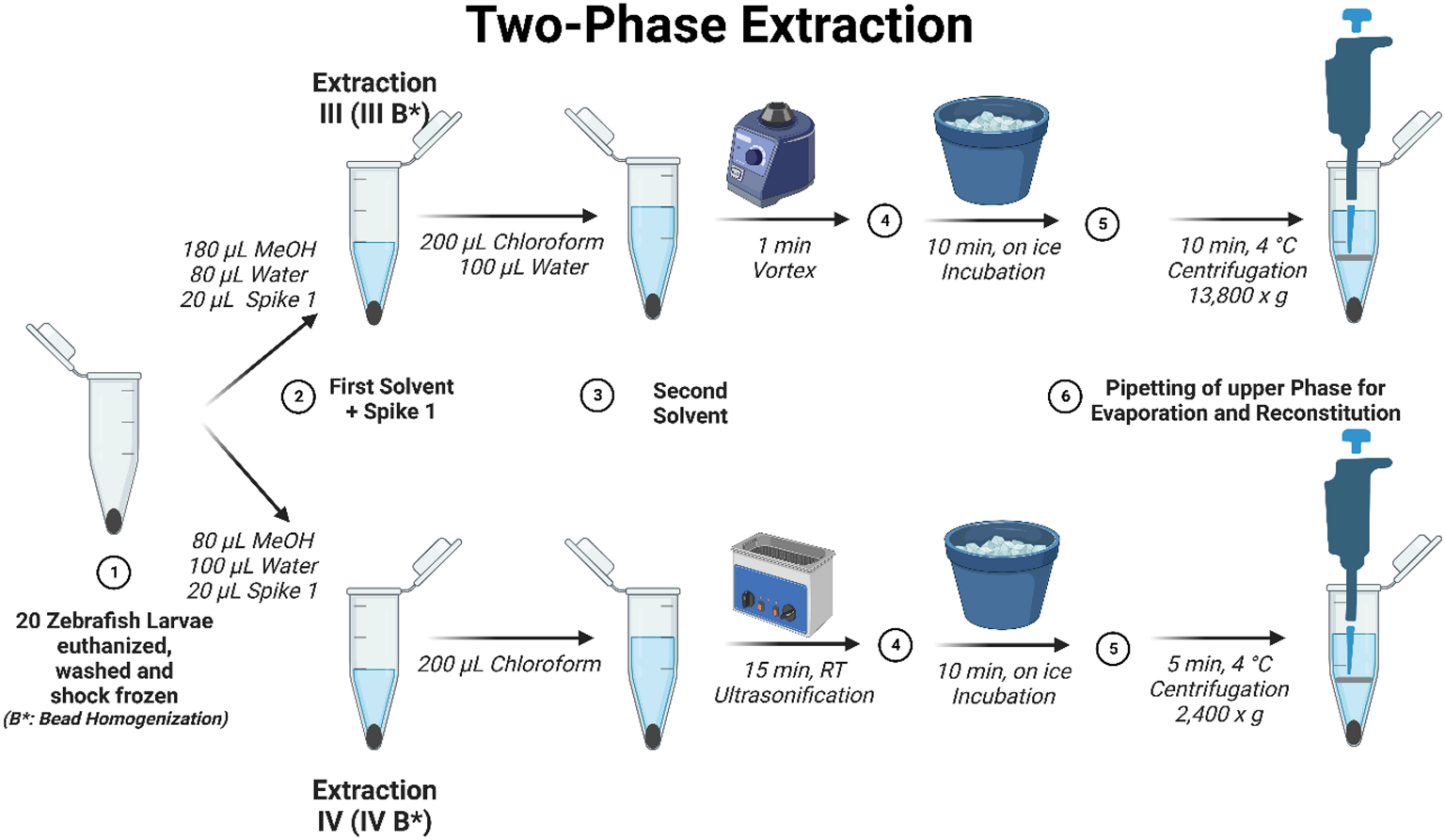
Workflow for extraction procedures III (III B) and IV (IV B). *MeOH* Methanol, *ACN* Acetonitrile. *Spike 1 solution* 25 µg/mL tryptophan-d_5_, 1 mg/mL palmitic acid-d_31_, 50 µg/mL glucose-d_7_ in MeOH. Generated by BioRender.com and published based on their academic license terms.

For extractions IV and IV_B, adapted from Ding et al., 80 µL of MeOH and 100 µL of Millipore water, together with 20 µL spike solution 1 were added to each sample^20^. 200 µL chloroform were added and the samples were sonicated using an USC 100T ultrasonic cleaner (VWR, Darmstadt, Germany) for 15 min. The samples were incubated on ice for 10 min and afterwards centrifuged at 4 °C and 2400×*g* for 5 min. The workflows of each individual extraction procedure can be found in Figs. 1 and 2.

For the extractions III, IV, III_B, and IV_B the lower, lipophilic phase was discarded and not further analyzed because the analytical setup including ESI ionization was not optimized for lipophilic metabolites, as well as most molecules of interest and internal standards being hydrophilic.

After each extraction procedure, the supernatant of each sample was transferred into brown glass vials and kept at − 80 °C overnight. A volume of 10 µL spike solution 2 (1 µg/mL trimipramine-d_3_ and 50 µg/mL glibenclamide in MeOH) was added, and the solvent was removed using a vacuum centrifuge (Eppendorf, Hamburg, Germany) at 30 °C, using the setting for alcoholic solutions under vacuum (V-AL) for up to 2 h, depending on the solvent volume. Afterwards, the residue was reconstituted in 50 µL MeOH/ACN (70:30 *v/v*), containing 0.01 µg/mL bisoprolol-d_5_ and telmisartan-d_7_, as well as 10 µg/mL hydrochlorothiazide and furosemide (spike solution 3). The reconstituted extract was vortexed for 10 s, and each sample was transferred into an autosampler inlet. Volumes of 5 µL of each sample were pooled and used as a QC sample. Samples were maintained on dry ice for transport between facilities and stored at − 80 °C until analysis.

### LC-HRMS/MS analysis

The LC-HRMS/MS analysis was conducted based on previous publications^36,38^ and details about the instrumentation can be found in the supplementary material. Briefly, the used apparatus consisted of a Thermo Fisher Scientific Dionex UltiMate 3000 RS pump, including degasser, quaternary pump, and UltiMate Autosampler, coupled to a TF Q-Exactive Plus (TF, Dreieich, Germany). The ion source was a heated electrospray ionization HESI-II source. The parameters for the HESI-II source were chosen according to a previous method^36^.

Reversed phase (RP) chromatography was performed on a TF Accucore Phenyl-Hexyl column (100 mm × 2.1 mm, 2.6 μm, TF, Dreieich, Germany) and hydrophilic interaction liquid chromatography (HILIC) using a Nucleodur column (125 mm × 3 mm, 3 μm, Macherey-Nagel, Düren, Germany).

### Data processing and statistical analysis

TF raw files were analyzed using TF Xcalibur software version 4.1 for retention time, peak intensity, and peak shape for all IS and MoInt to assure correct detection, identification, and possible detector saturation. Afterwards, the raw files were converted into mzXML files using ProteoWizard^39^. Peak picking was performed using XCMS in an R environment^40^. Additionally, detected peaks were annotated as isotopes, adducts, and artifacts using the CAMERA package^41^. The optimization of XCMS parameters was done based on a previously published method^42^ The optimized peak picking and alignment parameters can be found in Table S2. After peak picking and grouping across samples into individual features, the feature areas were batch-corrected using the repeated measurement of the pooled QC samples^43^.

The feature count was determined and analyzed using one-way analysis of variance (ANOVA) and Welch’s two-sample *t* test based on a previously published method^42^ using XCMS, comparing extraction II, III and IV against I, III against III_B and IV against IV_B respectively.

Based on the peak areas of internal standards (IS) and MoInt, taken from the evaluation script, values for mean, standard deviation, and CV were calculated using excel and diagrams were constructed using GraphPad PRISM 9. Additionally, based on Manier and Meyer^42^, the peak areas of the internal standards as well as the MoInt were analyzed using ANOVA and Welch’s two-sample *t* test, comparing every extraction against extraction I. Extraction I was considered as the reference method, as previously successfully used for other metabolomics studies^19^, as well as in preliminary experiments.

ANOVA was applied to the whole generated dataset to determine significantly different molecules or features between the six different sample groups, employing Bonferroni correction^44^ to correct for false positive results. To investigate group wise differences between extraction methods, principal component-discriminant function analysis (PC-DFA) was conducted using the significant features. Prior to principal component analysis, the dataset was centered, and the subsequent discriminant analysis used those components that fulfilled Kaiser’s criterion with a minimum of two components. Prediction quality was determined using Monte Carlo cross-validation^45^.

The raw data files are uploaded to MetaboLights (https://www.ebi.ac.uk/metabolights/) with the study identifier MTBLS6046. The evaluation script for data preprocessing and the statistical analysis in R can be found on Github (https://github.com/PhilSchip/zebrafish_extraction).

### Identification of molecules of interest (MoInt)

The MoInt included 59 molecules, mainly host metabolites that were found significantly altered in mycobacterial infection models using zebrafish and other species, as well as metabolites from preliminary infection experiments done prior to this study as a form of a pseudo-targeted approach. Metabolites from one additional study unrelated to infection research^37^, were included, due to the adaption of the extraction method in this paper.

Based on the *m/z* and retention time, parallel reaction monitoring was conducted with the pooled QC sample to identify the MoInt with an identification level of 2 as putatively annotated compounds instead of being classified as unknown compounds, using a classification system proposed by Sumner^46^. Data files were converted to the mzXML format using ProteoWizard^39^ and imported into NIST MS Search 2.3. Three different databases were used for compound identification: Human Metabolome Database (hmdb), NIST14 (nist_msms and nist_msms2 subdatabases), and the Wiley METLIN Mass Spectral Database (metlin_insilico and metlin_experimental subdatabases). The following parameters, based on a previous publication^36^ were used for the library search: spectrum search type, identity (MS/MS); precursor ion *m/z*, in spectrum; pre-search, off. The MS/MS parameters were as follows: settings: precursor tolerance,  ± 5 ppm; product ion tolerance, ± 10 ppm.

### Pathway analysis of molecules of interest

To determine the biological relevance of the detected molecules of interest, pathway analysis using version 5.0 of Metaboanalyst^47^ was conducted. HMDB IDs of each MoInt were used as identifiers, connected to their corresponding KEGG identifiers, and analyzed against the Danio Rerio pathway library of KEGG^48–50^. With 3-ketocholesterol having no HMDB ID and 3-methoxytyrosine, stearoyl ethanolamide and dimethylarginine having no KEGG ID, 31 could be used for this analysis. The analysis was conducted using scatter plot as visualization method, hypergeometric test as enrichment method, relative-betweenness centrality for topology analysis, as well as using all compounds of the pathway library as reference metabolome.

## Results and discussion

### Impact of extraction on total feature count

**Figure 3:**
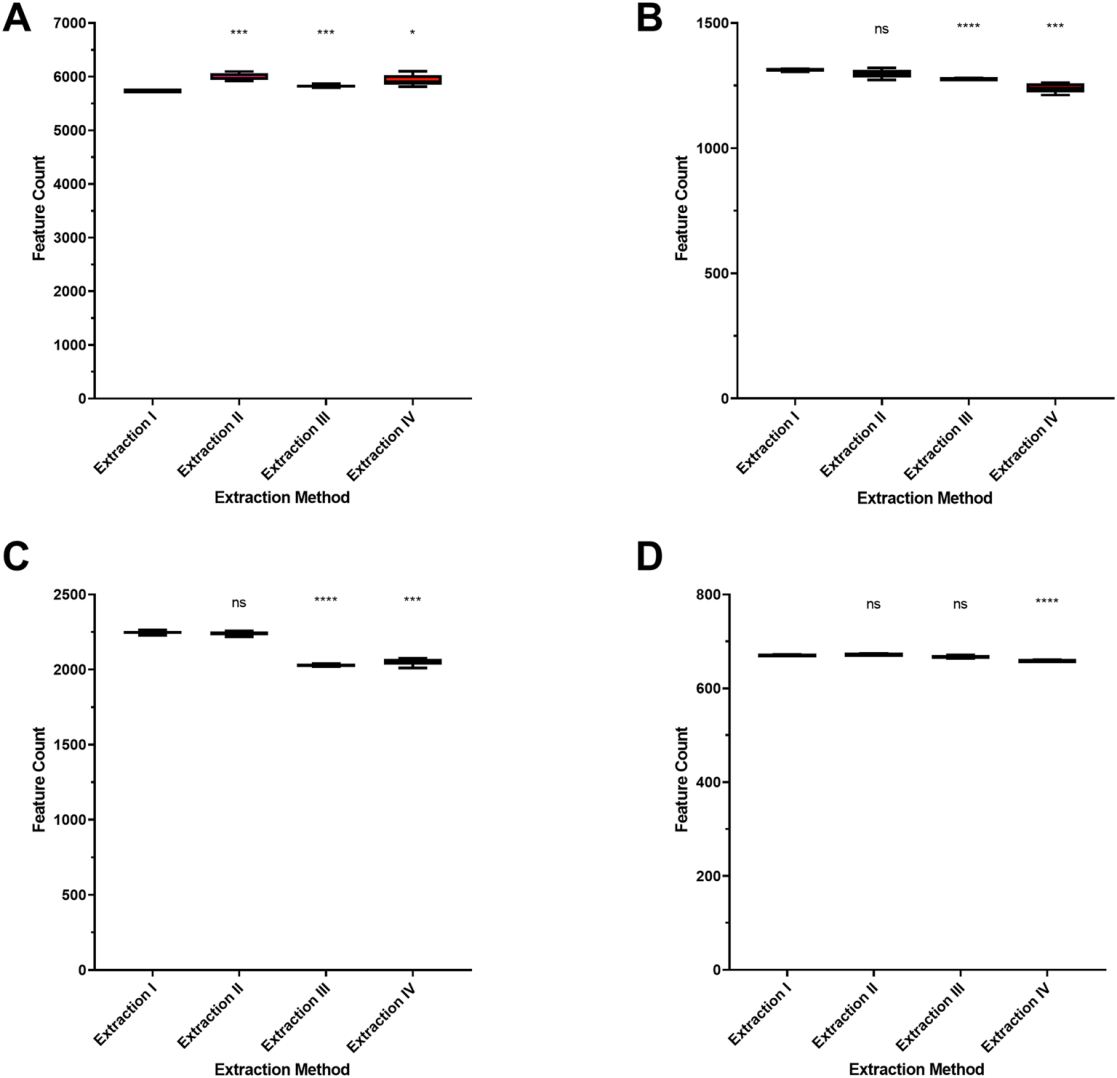
Number of detected features. Statistical evaluation was performed using one-way ANOVA and Welch’s two sample *t* test comparing each group to extraction I. (**A**) Phenyl-Hexyl column, positive ionization. (**B**) Phenyl-Hexyl column, negative ionization. (**C**) HILIC column, positive ionization. (**D**) HILIC column, negative ionization. ns not significant; *p < 0.05; **p < 0.01; ***p < 0.001; ****p < 0.0001. n = 5 for each sample group.

The four different extraction methods were first analyzed based on the feature count. While the feature count as an analyzed parameter of quality is of lower relevance in targeted approaches, it can be a useful surrogate for untargeted metabolomics. While it does not affect the results of the pseudo-targeted approach, it correlates with the coverage of the whole metabolome better and thus provides a higher chance of detecting wanted, but untargeted metabolites. As shown in Fig. 3, extractions I and II allowed the detection of more features, compared to the two-phase methods III and IV. Due to the loss of lipophilic metabolites during the phase separation, this observation was expected, and could be circumvented using a dedicated method for lipophile substances on the discarded lipophilic extract. The only exception was the analysis on the Phenyl-Hexyl column using positive ionization mode, were II, III and IV were all increased. For extraction II and IV the ultra-sonification and therefore higher homogenization of the samples could explain this effect, which is not true for extraction III. Looking at the other runs, no such effect of the ultra-sonification could be observed. Therefore, no explanation is possible as of now.

Apart from this exception, extractions I and II showed a very similar feature count, while methods III and IV differed, based on the polarity of the analysis. Extraction IV showed higher feature counts after positive ionization (A and C), and extraction III after negative ionization (B and D), respectively.

In general, positive ionization mode allowed the detection of more features than negative ionization mode, with around 2200–6000 features compared to around 600–1300 features, respectively. It was also observed that analysis after using the HILIC column showed less than half of the features than analysis after using the Phenyl-Hexyl column.

**Figure 4:**
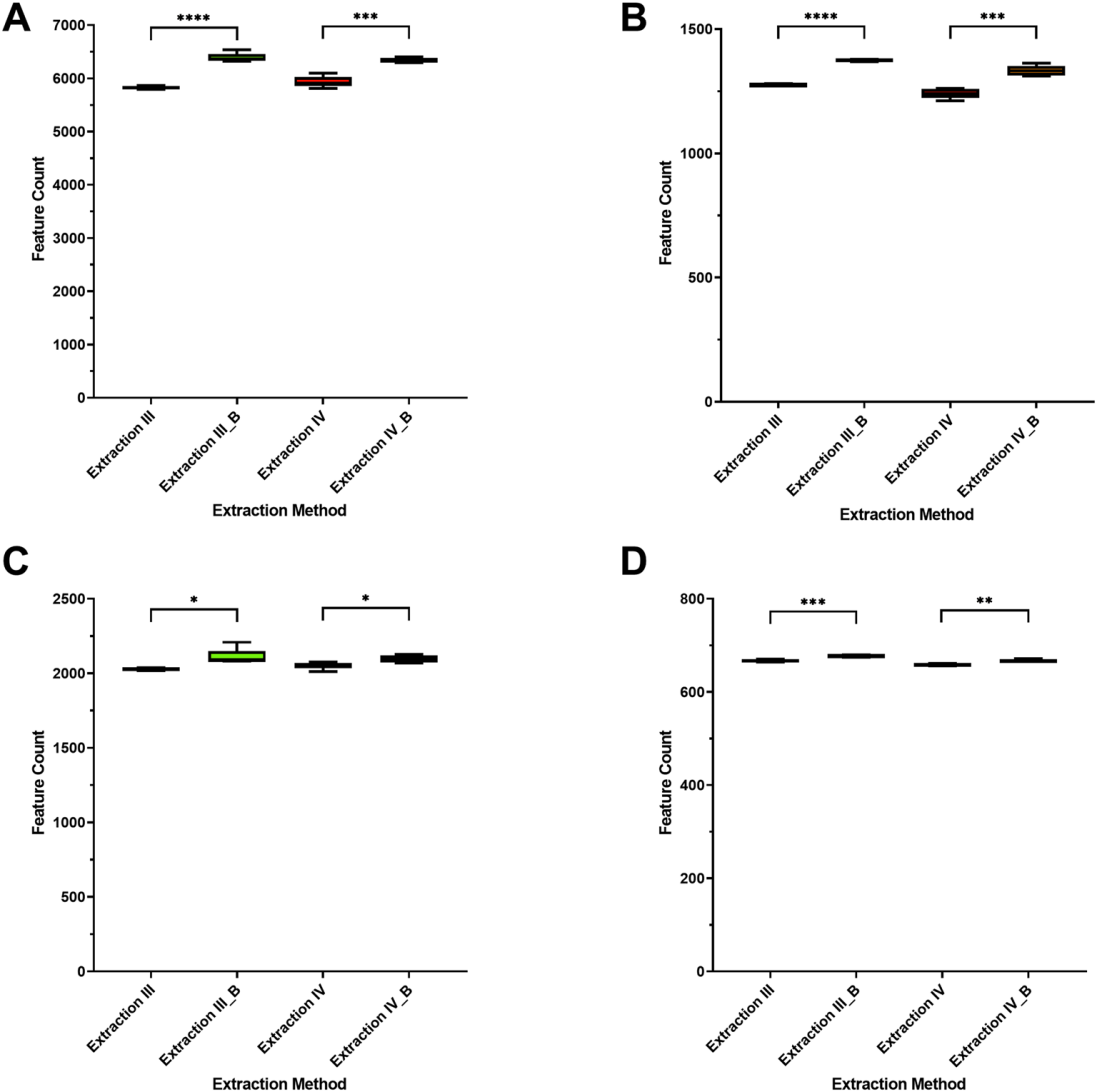
Number of detected features. Statistical evaluation was performed using one-way ANOVA and Welch’s two sample *t* test comparing each group to extraction I. (**A**) Phenyl-Hexyl column, positive ionization. (**B**) Phenyl-Hexyl column, negative ionization. (**C**) HILIC column, positive ionization. (**D**) HILIC column, negative ionization. *ns* not significant; *p < 0.05; **p < 0.01; ***p < 0.001; ****p < 0.0001. n = 5 for each sample group. Addition of bead homogenization was denoted as “_B”.

Using mechanical pre-treatment led to a significant increase in feature count (Fig. 4) in all conducted runs. This indicates the successful homogenization of the samples, which leads to more broken-down larvae tissue and more metabolites, released for the extraction.

Based on these results, two-phase methods missed features, most likely due to the separation from hydrophilic and lipophilic substances and the subsequent discarding of those lipophilic substances. While the extracts itself should contain fewer interfering molecules, the possibility of missing relevant features is problematic. For the use of bead homogenization, the contrary was shown. A higher feature count, equal or higher in some cases than the one-phase extractions led to a better coverage, with a higher chance of interfering molecules.

Both results must be treated cautiously, nonetheless. Both, endogenously derived signals, for example resulting from isotopes, adduct formation or fragmentation, as well as artificial signals from contaminants, noise, or errors in data processing^51^ can lead to a significant variation in measured feature count.

However, in this study, the low variability in each sample group, as well as the compensation of random erroneous signals by five repeated extractions per group, lead to high confidence in these results. While those variations could still play a role in the observed differences, those being a major part seems unlikely.

These results indicate differences between each extraction principle, while methods using the same principle showing much closer results across all features, as expected. If these differences are relevant for the future infection model or impact only molecules irrelevant for the investigation of *M. marinum* infection is unknown as of now. To further evaluate the differences and their relevancy, internal standards and MoInt, selected based on the scientific context of a future application of the extraction procedure for an *M. marinum* infection model, were analyzed in the following steps.

### Impact of extraction on internal standards

The used internal standards can be divided into three separate groups based on their addition at the beginning of the extraction (spike 1), prior to evaporation (spike 2), or during the reconstitution (spike 3). Their peak picking success depending on the used column and ionization as well as their retention time and *m/z* can be found in Table S1. Noticeable is the fact that tryptophan-d_5_ and glucose-d_7_ were not detected using HILIC column and negative ionization mode, most likely due to very low intensities, and in the case of glucose-d_7_ additionally, due to an uncharacteristically wide peak shape. The lower intensities of the IS after negative ionization, especially after HILIC could not be circumvented. The peak shape of glucose-d_7_ after HILIC indicated the need for an optimization of the chromatography, which was not done in the context of this study due to the low relevance of saccharides in the MoInt, as well as the sufficient quality of detection after RP chromatography.

The peak areas of each detected internal standard were normalized on the mean peak area using extraction I, used as reference standard due to being an already established method. These normalized peak areas can be found in Figs. S2–S5. The respective CV values can be found in Figs. S6–S9. Additionally, Welch’s two sample *t* tests were conducted, comparing the peak areas of extractions II, III, and IV with extraction I. The resulting significant differences are summarized in Tables S3–S6.

The peak areas of tryptophan-d_5_ were very similar between the extraction methods, except for extraction IV, which showed a notable decrease in all three analytical runs.

Glucose-d_7_ could only be detected using Phenyl-Hexyl column and negative ionization mode, and very similar peak areas, except an increase for extraction II, were observed.

Palmitic acid-d_31_ showed a significant difference between one-phase and two-phase methods. Most likely due to its high lipophilicity as a long-chain fatty acid, the recovery using two-phase methods was almost nonexistent, while extractions I and II were equally able to extract palmitic acid-d_31_.

Using negative ionization, apart from lower peak areas for glibenclamide on the HILIC column for extraction II, III and IV, the peak areas of the other six tested internal standards of spikes 2 and 3 were very similar between extractions. For positive ionization, however, the trend was observed for glibenclamide and trimipramine-d_3_ on the Phenyl-Hexyl column and for the same IS, as well as bisoprolol-d_5_ and telmisartan-d_7_, with extraction IV having the lowest observed peak areas. Those IS were added just before the evaporation and reconstitution, those differences can only originate from this point onward. Besides interference from other molecules, the longer duration of vacuum evaporation could be a factor for the loss of intensity. On the other hand, the same effect was observed for bisoprolol-d_5_ and telmisartan-d_7_ contained in spike solution 3, which were added in the reconstitution solution, but only prior to HILIC-based analysis. Therefore, the interaction of those IS with other molecules during the LC run or adhesion to the non-silanized brown glass vials used were more likely.

Regarding the CVs, the majority were in between 5 and 20%. Exceptions include palmitic acid-d_31_ for two-phase extractions due to the very low peak areas, glucose-d_7_ for extraction IV, as well as glibenclamide on the HILIC column and bisoprolol-d_5_, using extraction II.

Overall, all extraction methods showed relatively low CV values, with extraction II having the worst precision and extraction III being preferred ahead of extraction IV based on the recovery of tryptophan-d_5_.

Besides a significant increase of furosemide peak areas (negative ionization, Phenyl-Hexyl column), no significant changes in the peak areas of the IS could be observed. Mechanical pre-treatment led to an increase of CVs for tryptophan-d_5_ across the board, except for extraction IV_B on the HILIC column (positive ionization), which showed a reduction of ~ 2% points, while glucose-d_7_ showed an increase using method III and a decrease using method IV in the same order of magnitude.

### Impact of extraction on molecules of interest

To further evaluate the differences between extraction methods that could be relevant for the chosen analytical problem, a comprehensive library of so-called MoInt was used. In total, 59 MoInt were selected for evaluation, based on literature and preliminary in-house experiments, which can be found in Table S7. After detection and manual evaluation of the resulting peaks, 34 and 16 molecules of this list remained, using positive and negative ionization, respectively. Calibration ranges are usually tested beforehand for quantitative and targeted approaches to ensure the analysis of target molecules within the dynamic range of a concentration–response curve. This approach is not feasible for untargeted approaches, due to the wide range of possible and unknown targets. This study and the further planned infection studies are employing a qualitative approach, only comparing relative amounts of the MoInt between different sample groups without quantifying the concentration of the target molecules. Nevertheless, detector saturation can be an issue even in untargeted and qualitative studies. To evaluate this effect in the context of the current study, the peak shapes and intensities of all MoInt and IS where analyzed manually using the TF Xcalibur software. Signals were evaluated for signs of saturation, such as irregular, flattened peak shapes, or particularly high signal intensities. None of these effects were observed within the data of this study. The resulting data should therefore be considered robust, regarding the analyzed MoInt and IS, while it has to be acknowledged that for future untargeted metabolomic studies, saturation effects should be excluded by additional dilution experiments and experiments with higher amounts of substrate (in this case zebrafish larvae).

The list of these successfully detected MoInt, their compound subgroup, as well as their detection on each column, inclusion parameters, and identification status based on spectra generated in follow-up PRM runs of the pooled QC sample, can be found in Table 1 for positive ionization mode and Table 2 for negative ionization mode.

**Table 1:** Experimental parameters of all used molecules of interest for positive ionization including detection using PRM runs, mass-to-charge ratio (*m/z*) and retention time (rt) for reversed-phase (RP) and hydrophilic interaction liquid chromatography (HILIC) column, as well as the compound sub class based on the HMDB database. Exclusion was based on overall peakshape and intensity. *n.d.* not detected.

Compound name	Identification		Detection		*m/z*	rt [s]		Compound sub class
	RP	HILIC	RP	HILIC		RP	HILIC	
2-Aminobenzoic acid	×	×	✓	✓	138.055	30	447	Benzoic acids
3-Dehydroxycarnitine	✓	×	✓	×	146.118	28	567	Fatty acids
3-Ketocholesterol	✓	×	✓	×	385.347	588	74	Cholestane steroids
3-Methoxytyrosine	✓	×	✓	×	212.092	58	0	Amino acids
Adenosine	✓	✓	✓	✓	268.104	59	298	Purine nucleosides
Aminoadipic acid	×	×	✓	×	162.076	36	461	Amino acids
Arginine	✓	✓	✓	✓	175.119	23	556	Amino acids
Asparagine	×	×	✓	×	133.061	23	517	Amino acids
Aspartic acid	✓	×	✓	×	134.045	25	542	Amino acids
Creatine	✓	✓	✓	✓	132.077	25	511	Amino acids
Cytidine	✓	✓	✓	✓	244.093	27	414	Pyrimidine nucleosides
Dimethylarginine	×	✓	×	✓	203.150	23	554	Amino acids
Glutamic acid	✓	✓	✓	✓	148.060	25	538	Amino acids
Glutamine	✓	✓	✓	✓	147.076	23	515	Amino acids
Glycerophosphocholine	✓	✓	✓	✓	258.110	24	540	Glycerophosphocholines
Histidine	✓	×	✓	×	156.077	21	513	Amino acids
Hypoxanthine	✓	✓	✓	✓	137.046	35	303	Purines
Inosine	✓	✓	✓	✓	269.088	56	361	Purine nucleosides
Inosinic acid	✓	✓	✓	✓	349.054	29	543	Purine ribonucleotides
Leucine	×	✓	✓	✓	132.102	41	456	Amino acids
Lysine	×	✓	✓	✓	147.113	22	562	Amino acids
Methionine	✓	✓	✓	✓	150.058	32	468	Amino acids
*N6*,*N6*,*N6*-trimethyl-l-lysine	×	×	✓	×	189.160	22	602	Amino acids
Nicotinamide	✓	×	✓	×	123.055	45	170	Pyridinecarboxylic acids
*N*-Palmitoyl-d-Sphingosine	×	×	✓	×	538.519	602	85	Ceramides
Phenylalanine	✓	×	✓	×	166.086	77	448	Amino acids
Pipecolic acid	×	×	✓	×	130.086	23	192	Amino acids
Propionylcarnitine	×	✓	✓	✓	218.139	288	484	Fatty acid esters
Stearoylethanolamide	✓	×	✓	×	328.322	506	91	Amines
Threonine	×	×	✓	×	120.066	24	509	Amino acids
Tryptophan	✓	✓	✓	✓	205.097	139	452	Indolyl carboxylic acids
Tyrosine	✓	✓	✓	✓	182.081	35	475	Amino acids
Uridine	×	×	✓	×	245.077	31	251	Pyrimidine nucleosides
Xanthine	✓	✓	✓	✓	153.041	37	269	Purines

**Table 2:** Experimental parameters of all used molecules of interest for positive ionization including detection using PRM runs, mass-to-charge ratio (*m/z*) and retention time (rt) for reversed-phase (RP) and hydrophilic interaction liquid chromatography (HILIC) column, as well as the compound sub class based on the HMDB database. Exclusion was based on overall peak shape and intensity.

Compound name	Identification		Detection		*m/z*	rt [s]		Compound sub class
	RP	HILIC	RP	HILIC		RP	HILIC	
Aminoadipic acid	×	×	×	✓	160.062	36	461	Amino acids
Arginine	×	×	×	✓	173.104	23	556	Amino acids
Cytidine	×	×	×	✓	242.078	27	414	Pyrimidine nucleosides
Glucose	×	×	✓	×	179.056	26	475	Carbohydrates
Glutamic acid	✓	✓	✓	✓	146.05	25	538	Amino acids
Glutamine	✓	✓	✓	✓	145.062	23	515	Amino acids
Histidine	✓	×	✓	×	154.062	21	513	Amino acids
Hypoxanthine	✓	✓	✓	✓	135.031	35	303	Purines
Inosine	✓	✓	✓	✓	267.073	56	361	Purine nucleosides
Inosinic acid	✓	✓	✓	✓	347.040	29	543	Purine ribonucleotides
Methionine	×	×	✓	✓	148.044	32	468	Amino acids
Phenylalanine	✓	✓	✓	✓	164.072	77	448	Amino acids
Tryptophan	✓	×	✓	×	203.083	139	452	Indolyl carboxylic acids
Tyrosine	✓	×	✓	✓	180.066	35	475	Amino acids
Uridine	✓	✓	×	✓	245.078	31	262	Pyrimidine nucleosides
Xanthine	✓	✓	✓	✓	151.026	37	269	Purines

Like the peak areas of the internal standards (see Sect. 3.1.2), the peak areas of each detected MoInt were normalized on the mean peak area of extraction I and the means for each extraction, as well as their standard deviations, can be found in Figs. S10–S13. The results of Welch’s two sample *t* test, comparing extractions II, III, and IV with extraction I can be found in the Tables S8–S11.

In all four analytical runs, most MoInt were detected with peak areas roughly equal to that for extraction I, especially for extraction II, being the most closely related method to extraction I. Using two-phase extraction methods led to a significant reduction of peak areas for a few MoInt, most notably 3-Ketocholesterol, *N*-Palmitoyl-d-Sphingosine, and inosinic acid. In contrast, a wide range of molecules, especially amino acids, were increased significantly. While the lower recovery can be explained based on the lipophilicity of the affected MoInt, the increased peak areas for amino acids could be attributed to multiple factors, e.g., overall higher recovery, higher number of interfering signals for similar *m/z* and retention time values, or lower amounts of molecules, interfering with the ionization of the MoInt.

Especially a higher number of interfering molecules could play an important role, when using the Phenyl-Hexyl column, due to most amino acids eluting very early inside the first 60 s.

**Figure 5:**
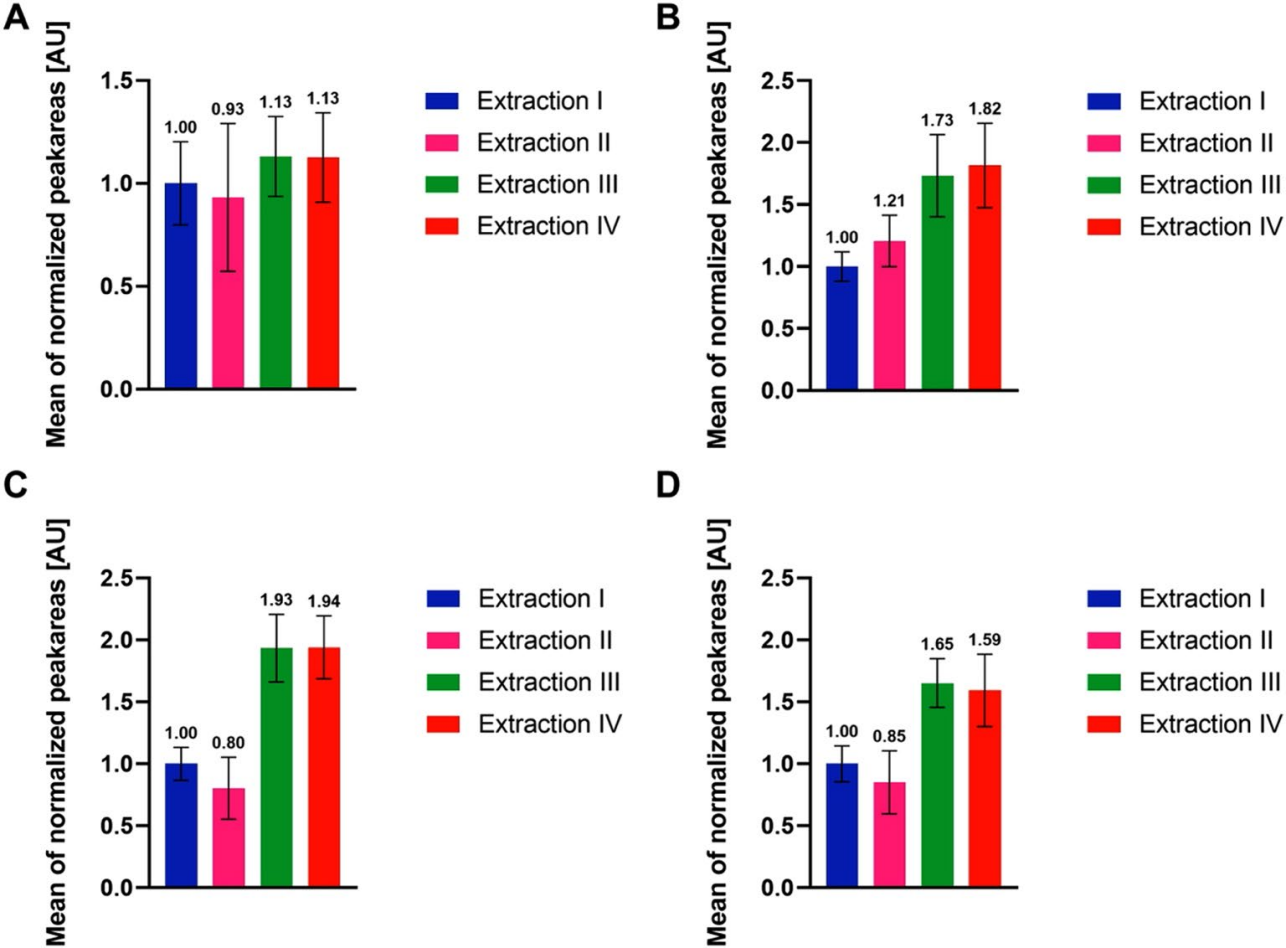
Mean of normalized peak areas of each extraction procedure and their respective standard deviation. n = 5 for each sample group. (**A**) Phenyl-Hexyl column, positive ionization. (**B**) Phenyl-Hexyl column, negative ionization. (**C**) HILIC column, positive ionization. (**D**) HILIC column, negative ionization.

In summary, two-phase extractions seemed to have the advantage of higher MoInt recovery, with both methods III and IV showing very similar results. Regarding single-phase extractions, extraction I seems to be the most promising, with respect to feature recovery. Summarizing those findings for all MoInt for each extraction (Fig. 5) showed the single-phase methods with overall much lower peak areas, with extraction II showing the lowest recovery, except on the Phenyl-Hexyl column using negative ionization. Extractions III and IV showed very similar results. Looking at the standard deviation extraction I showed the highest precision, while extraction II had lower precision across the board, and both two-phase methods showed the highest standard deviations and lowest precision, except extraction II on the Phenyl-Hexyl column and positive ionization.

To visualize the precision of each individual MoInt, respective CV values are shown in Figs. S14–S17. Most CVs were between 5 and 25%. Utilizing positive ionization leads to more precise results, compared to negative ionization, at least in part due to the overall lower intensities for most analytes in the negative runs, especially those detected via both positive and negative ionization. Extraction II showed on average the highest CV values and the most molecules with very high deviations, while extractions III and IV showed CV values mostly on par or slightly higher compared to extraction I. Some of the higher CV values were furthermore influenced by the previously mentioned lower recovery of lipophilic molecules. Extraction I showed the most precise results for single-phase extractions, while extraction III showed slightly lower CV for negative ionization and extraction IV for positive ionization, respectively.

**Figure 6:**
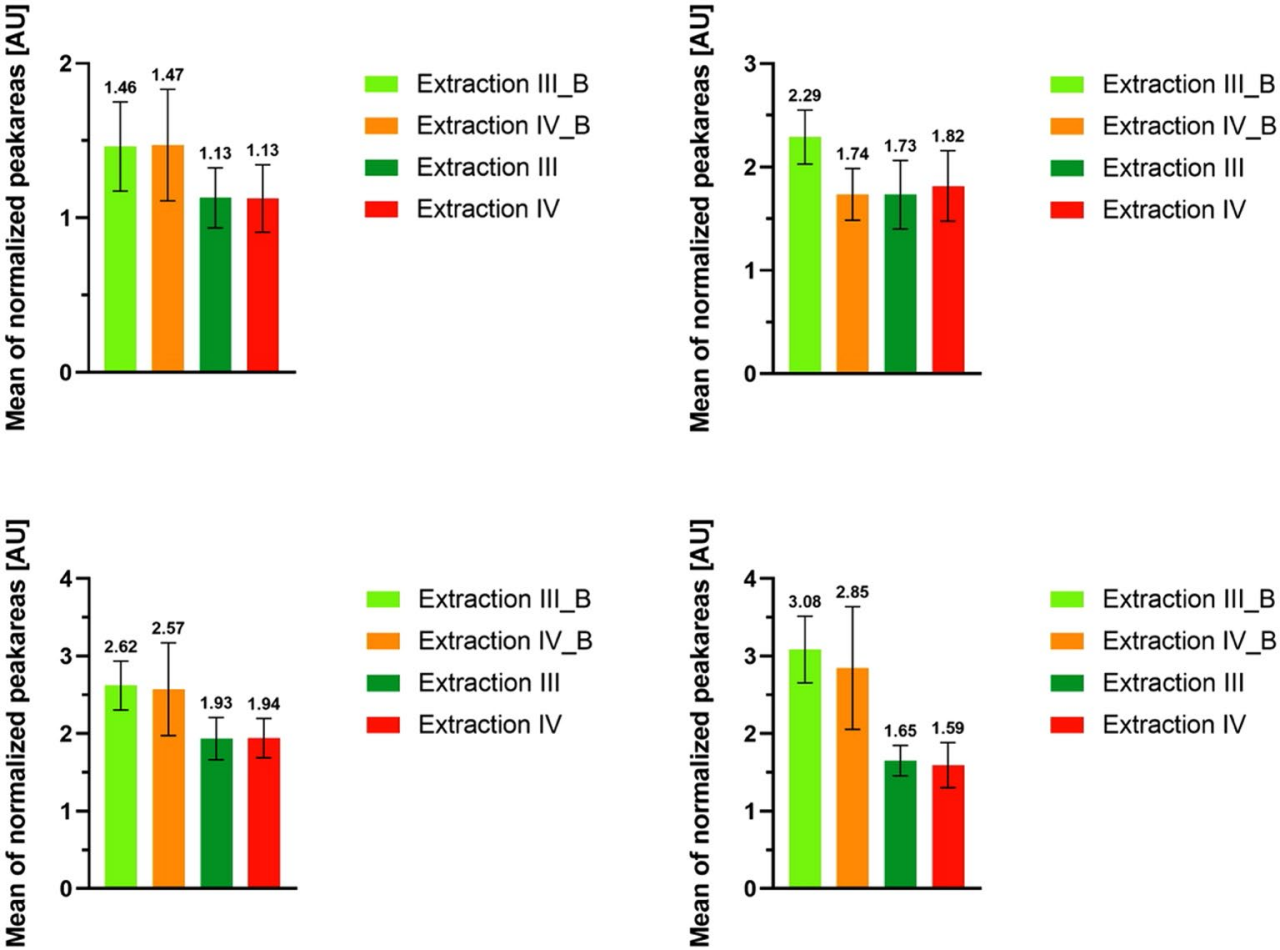
Mean of normalized peak areas of each extraction procedure and their respective standard deviation. n = 5 for each sample group. (**A**) Phenyl-Hexyl column, positive ionization. (**B**) Phenyl-Hexyl column, negative ionization. (**C**) HILIC column, positive ionization. (**D**) HILIC column, negative ionization. Addition of bead homogenization was denoted as “_B”.

The peak areas of extractions III and IV compared to their counterparts with added pre-treatment (extractions III_B and IV_B) can be found in Figs. S18–S21 of the supplementary material. Summarized in Fig. 6, both extractions with pre-treatment showed on average an increase in the MoInt peak areas. Extraction III_B showed higher peak areas for negative ionization and similar or slightly lower peak areas for positive ionization compared to IV_B. The higher peak areas were accompanied with higher or roughly equal standard deviations. The CVs of the individual MoInt showed no clear tendencies (Figs. S22–S25). Extraction IV_B led to higher CVs than III_B, and on average, the CVs were similar between respective extractions with or without the mechanical pre-treatment. In summary, bead homogenization increased the recovery of MoInt while keeping the precise equal to its counterpart with no added homogenization.

### Pathway analysis of molecules of interest

From the 35 MoInt detected in this study, 31 could be found in the KEGG database while the MoInt ketocholesterol, 3-methoxytyrosine, stearoyl ethanolamide and dimethylarginine were missing most likely to the fact that they are quite unexplored metabolites. 3-Ketocholesterol and stearoyl ethanolamide originated from preliminary in-house experiments and are therefore only tentatively linked to mycobacterium marinum infection in ZF, while 3-methoxytyrosine and dimethylarginine were linked to mycobacterial infection in ZF in another study^20^.

**Figure 7:**
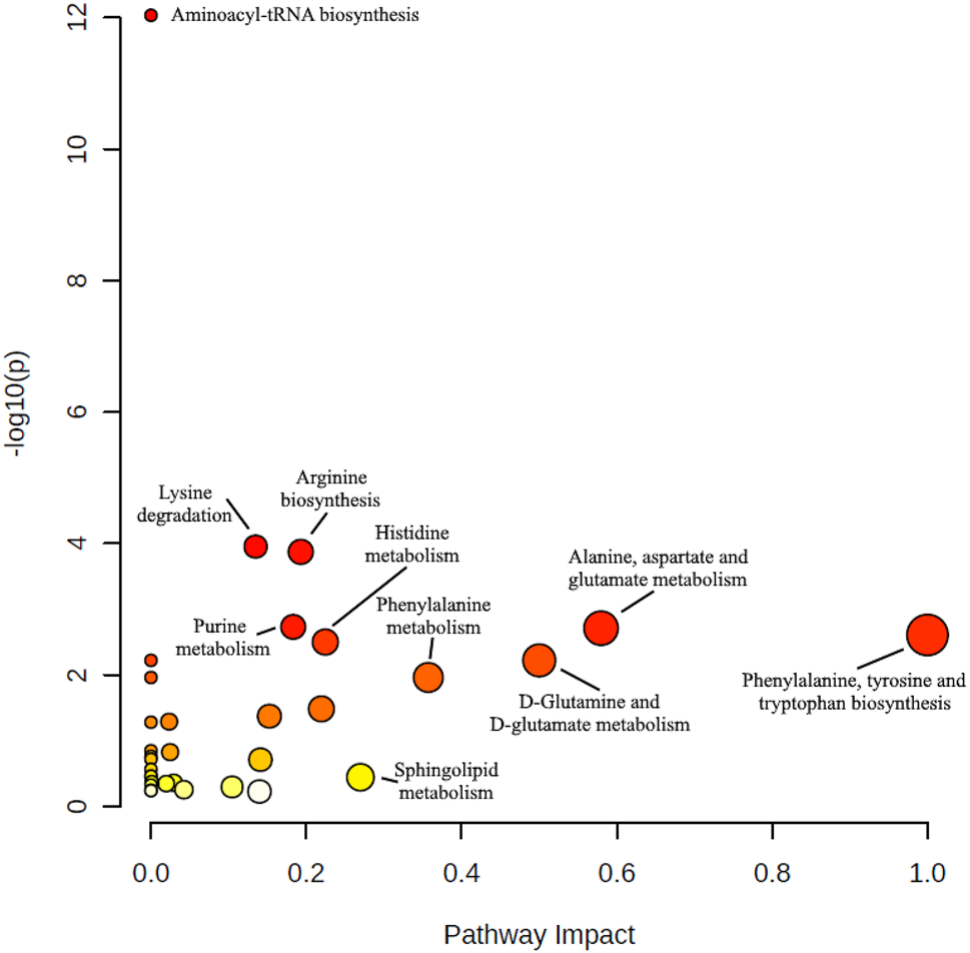
Summary of the pathway analysis of all detected MoInt, identifying the most relevant pathways by their impact and adjusted p values. Pathway impact was calculated as a combination of centrality and pathway enrichment, with the impact value positively correlating with the relative importance of a pathway. Circle colors indicate the significance (increasing from white to red) and circle sizes the pathway impact. Results were generated using Metaboanalyst version 5.0.

The summary of the pathway analysis for the MoInts can be found in Fig. 7. In total, 33 pathways were found with most of them being related to the metabolism of specific molecules e.g., metabolism of tryptophane, biotin, or phenylalanine. An overview of all pathways can be found in Table S12. Of those 33 pathways, nine showed a p-value of 0.01 or lower, indicating robust correlations of MoInt and the pathways. The aminoacyl-tRNA biosynthesis showed the highest amount of correlated MoInt. This is based entirely on the fact that the amino acid group is the largest group of detected MoInt and therefore as precursors of the aminoacyl-tRNA biosynthesis part of this pathway. However, the related impact score with 0.00 was very low. Much more relevant are pathways combining a high impact value and low p-values. Such pathways were the arginine biosynthesis and lysine degradation showing the lowest p-values after the tRNA biosynthesis and moderate impact, while the biosynthesis of phenylalanine, tyrosine, and tryptophan, as well as the metabolism of alanine, aspartate, and glutamate showed the highest impact values and are therefore most likely to be connected and relevant for other pathways and the metabolism as a whole.

In summary, the MoInt detected and analyzed in this study could be connected to several pathways of varying impact and, in combination with proven correlations with mycobacterial infections for most of them, the selected MoInt could be useful for future infection studies covering a variety of different metabolic pathways.

### Multivariate statistics

The whole metabolomes detected in each extraction procedure were analyzed using one-way ANOVA. Features that showed a significant difference between any two groups were kept for multivariate statistics. With 3016 and 638 significantly different features after analysis, using the Phenyl-Hexyl column, as well as 713 and 202 after analysis using the HILIC column for positive and negative ionization, respectively. Comparing these numbers to the previously discussed total feature counts, around 50% of all features were significant on the Phenyl-Hexyl column and around 30% on the HILIC column. This indicates that the Phenyl-Hexyl column is more effected by changes to the extraction procedure. Two possible effects could play a role in this phenomenon. First, different detectability of molecules, based on the separation on the different columns, leading to more molecules unaffected by the extraction being detected on the HILIC column or vice versa. Second, interactions between different molecules, e.g., quenching of ionization or interfering signals, which would be especially relevant in the first minute on the Phenyl-Hexyl column. Most amino acids in the MoInt, as well as many detected molecules were detected in this window, elevating the possibility of these interactions, and increasing the variability of a plethora of molecules. While a similar number of molecules could be directly affected by the extraction process, their co-elution on the Phenyl-Hexyl column could elevate their effect on the detected metabolome.

**Figure 8:**
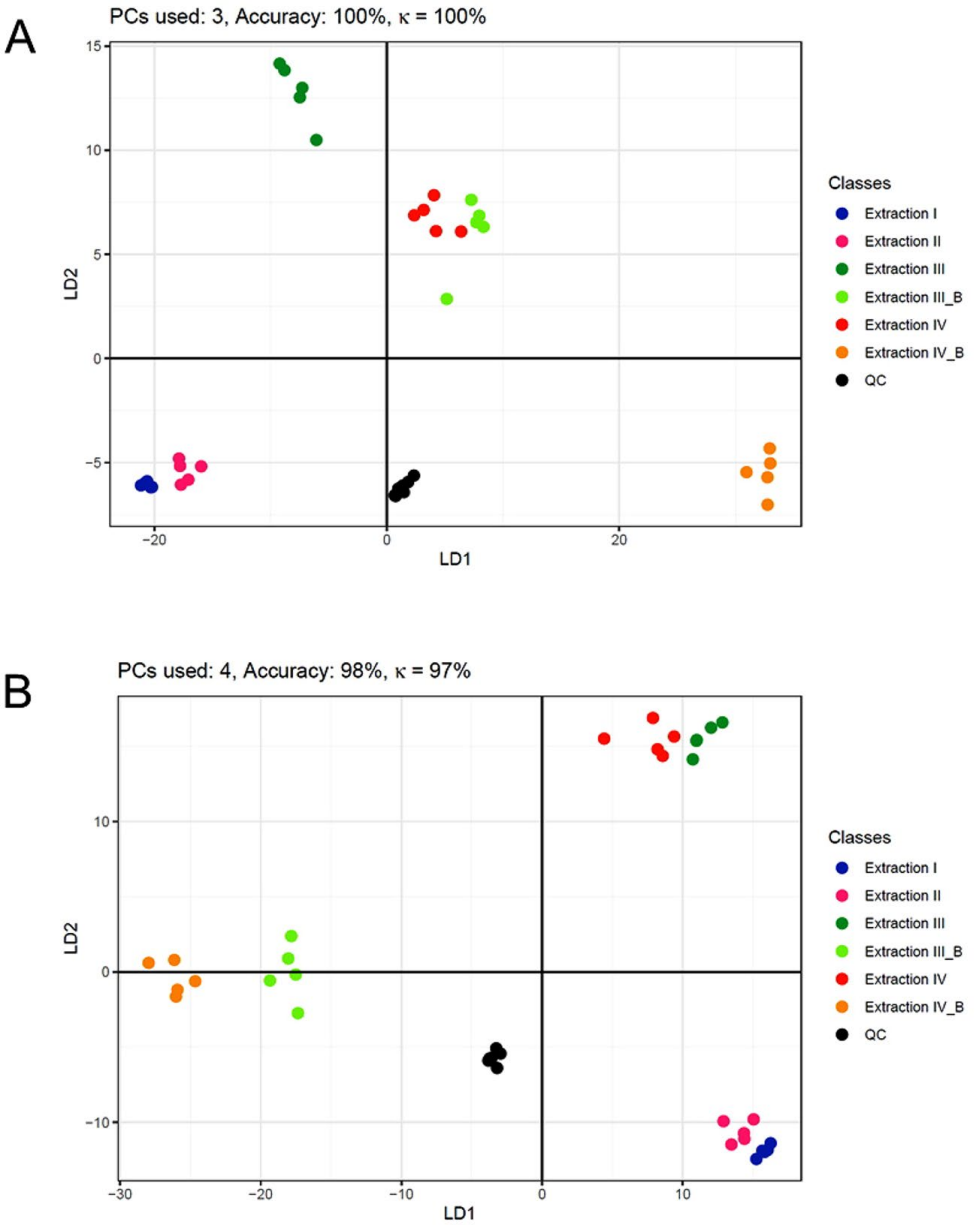
PC-DFA using significant features identified by one-way ANOVA. (**A**) Phenyl-Hexyl column, positive ionization mode. (**B**) Phenyl-Hexyl column, negative ionization mode. The number of used principal components, the prediction accuracy, and Cohen’s κ is provided. n = 5 for each sample group. Discriminant functions (DF1 and DF2) for maximizing between-class distance and minimizing within-class distance were plotted as x- and y-axis, respectively. Addition of bead homogenization was denoted as “_B”.

**Figure 9:**
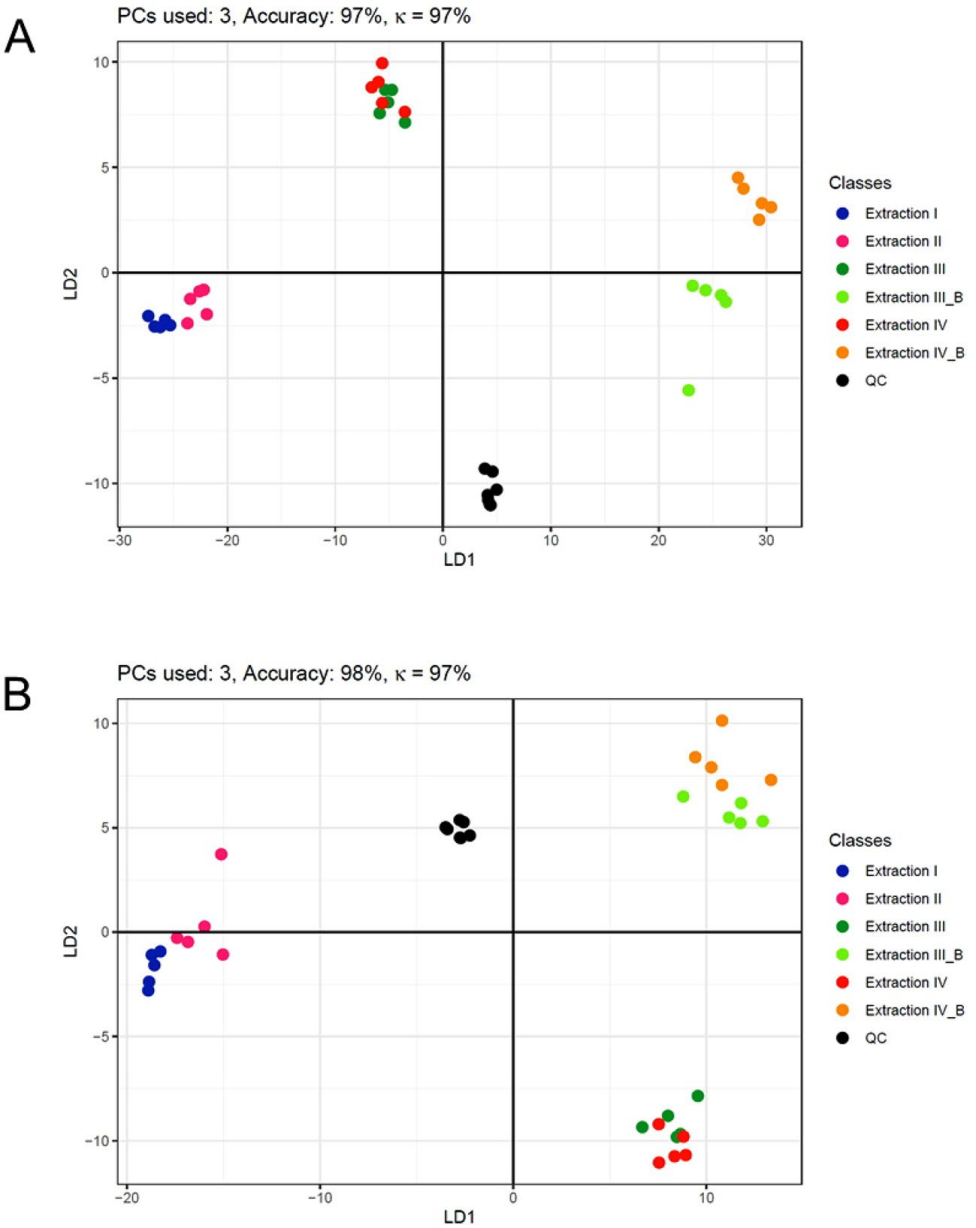
PC-DFA using significant features identified by one-way ANOVA. (**A**) HILIC column, positive ionization. (**B**) HILIC column, negative ionization. The number of used principal components, the prediction accuracy, and Cohen’s κ is provided. n = 5 for each sample group. Discriminant functions (DF1 and DF2) for maximizing between-class distance and minimizing within-class distance were plotted as x- and y-axis, respectively. Addition of bead homogenization was denoted as “_B”.

The following PC-DFA in Figs. 8 and 9 showed good separation between one-phase extractions (I and II), two-phase extractions (III and IV), and two-phase extractions with mechanical pre-treatment (III_B and IV_B). The only exception was found for the Phenyl-Hexyl column and positive ionization, with III_B and IV being very similar. This could indicate a similar effect of the bead homogenization of III_B and the ultra-sonification of IV, but this is not supported by the other results.

While extraction I and II, as well as III_B and IV_B were clearly separated in all runs, extraction III and IV were not clearly separated for the HILIC column, indicating a very similar coverage of the metabolome and thus no significant effect of the extraction differences between the two methods. Adding mechanical pre-treatment adds the separation, too, thus no consistent effect could be observed.

In summary, besides the previously mentioned exception, all six extractions showed different coverage of the whole metabolome, which indicated that the differences in extraction procedures have a different weight. Phase separation and bead homogenization seemed to change the extraction in a much more drastic manner than variations of other factors such as extraction solvents, incubation time or addition of ultra-sonification.

## Conclusions

Several differences could be observed amongst the tested subgroups (Table 3). Two-phase extractions without bead homogenization showed the lowest feature count, with both other groups having similar counts. Extraction II had the lowest recovery in this study, followed by extraction I. Both two-phase extractions had higher recovery rates, further increased by bead homogenization. The lowest CV values and therefore the best precisions were found for extraction I, with both two-phase extractions showed on average slightly higher CVs, further increased again after bead homogenization. Different extractions were compared in this study. One-phase extractions lead to the detection of more features overall and the feature areas of MoInt were lower in most cases compared to extractions III and IV, especially regarding amino acids. Only a few MoInt, as well as palmitic acid-d_31_, were reduced, most likely due to their lipophilicity and resulting removal in the lipophilic chloroform partition in two-phase extractions. The CVs of MoInt were lower for one-phase methods, especially extraction I, indicating a better overall precision and reproducibility. Additional larvae homogenization using glass beads increased the feature count and the recovery of MoInt, while slightly decreasing the precision. Thus, bead homogenization might be a possible optimization in the future if the advantages of higher recovery and feature count outweighs the more imprecise results. In summary, extraction I should be preferred over extraction II, based on overall higher peak areas of MoInt, lower CVs of those peak areas and similar feature counts, as well as an easier experimental process. Extraction III and IV showed similar peak areas and CVs for MoInt but extraction III showed a higher feature count, when using negative ionization, an easier experimental process, and higher peak area for tryptophane-d_5_.

**Table 3:** Summary of the results regarding feature count, peak areas, and coefficient of variation (CV) of all six compared extraction methods.

	Feature count	Peak areas	CV
Extraction I	↗	↘	↘
Extraction II	↗	↘↘	↗↗
Extraction III	↘	↗	↗
Extraction IV	↘	↗	↗
Extraction III_B	↗	↗↗	↗↗
Extraction IV_B	↗	↗↗	↗↗

Further experiments involving the infection model itself need to be performed, and both methods I and III, showing the best results in this study, need to be compared more in detail. For a more robust set of data increasing the number of used samples, repeated experiments, as well as the possible inclusion of other internal standards and additional MoInt might be added.

## Supplementary Information

Supplementary Information.

## Data Availability

Not all data generated during and/or analyzed during the current study are publicly available but are available from the corresponding author on reasonable request.
