# Supplementary material for: Evaluation of extraction methods for untargeted metabolomic studies for future applications in zebrafish larvae infection models
Source: Sci Rep. 2023 May 9;13:7489. doi: 10.1038/s41598-023-34593-y (PMC10170104; doi:10.1038/s41598-023-34593-y)
Supplement: Supplementary file 1 — Supplementary Information. [file 41598_2023_34593_MOESM1_ESM.pdf]

Evaluation of Extraction Methods for Untargeted Metabolomic Studies for Future Applications in Zebrafish Larvae Infection Models

Philip Schippers[1,2], Sari Rasheed[2,3], Yu Mi Park[2], Timo Risch[2,3], Lea Wagmann[1], Selina Hemmer[1], Sascha K. Manier[1], Rolf Müller[2,3], Jennifer Herrmann[2,3], and Markus R. Meyer[1]

[1]*Department of Experimental and Clinical Toxicology, Center for Molecular Signaling (PZMS), Institute of Experimental and Clinical Pharmacology and Toxicology, Saarland University, Homburg, Germany*

[2]*Helmholtz Institute for Pharmaceutical Research Saarland (HIPS), Helmholtz Centre for Infection Research (HZI), Saarland University, Saarbrücken, Germany*

[3]*German Centre for Infection Research (DZIF), Partner Site Hannover-Braunschweig, Germany*

Electronic Supplementary Material

Zebrafish Maintenance and Embryo Preparation

ZF husbandry and all experiments with ZF larvae were carried out in accordance with EU Directive 2010/63/EU and the German Animal Welfare Act (§11 Abs. 1 TierSchG). The ZF maintenance was based on previously documented methods (Westerfield 2007, Park, Meyer et al. 2020). In short, adult zebrafish for breeding were kept in an automated aquatic eco-system (PENTAIR, Apopka, UK). The following parameters are continuously monitored: Temperature (27 ± 0.5 °C), pH (7.0 ± 0.1), conductivity (800 ± 50 µs), and light-dark cycle (14 h/10 h). Fish were fed twice a day with dry small granulate food and additionally once a day with freshly hatched live Artemia cysts. For ZF embryo production, adult pairs of Casper ZF were kept overnight in standard mating cages, separated by gender. In the following morning, the separators were removed, and the zebrafish spawned immediately for two hours. The medium (0.3x Danieau's solution) consisted of 17 mM NaCl, 2 mM KCl, 0.12 mM $MgSO_4$, 1.8 mM $Ca(NO_3)_2$, 1.5 mM HEPES, and 1.2 µM methylene blue at a pH of 7.1–7.3. The ZF embryos at 1 dpf (days post fertilization) were dechorionated in a pronase solution (1 mg/mL in 0.3x Danieau's) for 6 min and then raised in an incubator at 28 °C until 5 dpf with a daily change of the medium. The timeline up to the euthanasia can be found in Figure S1.

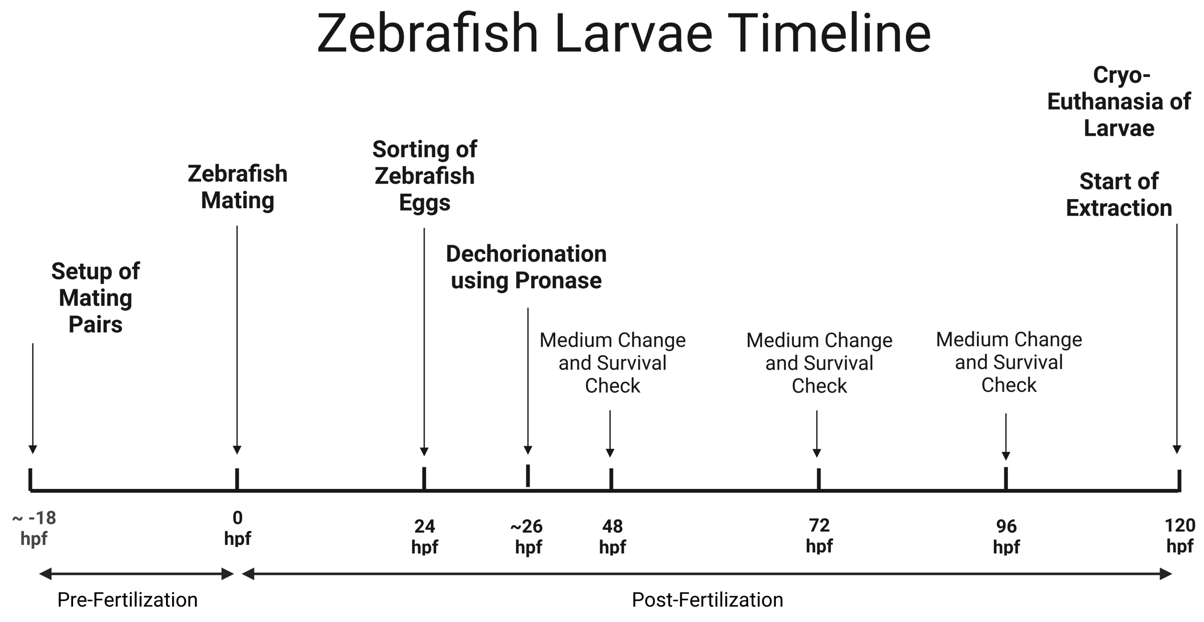

Figure S1. Timeline for the maintenance of zebrafish larvae. hpf = hours post fertilization. Generated by BioRender.com and published based on their academic license terms.

LC-HRMS/MS Analysis

The normal phase chromatography utilized a gradient of water (eluent C) with 200 mM ammonium acetate and acetonitrile (eluent D), containing 0.1% formic acid (*v/v*). The flow rate was set to 500 µL/min with the following gradient: 0-1 min 2% C, 1-5 min to 20% C, 5-8.5 min to 60% C, 8.5-10 min hold 60% C, 10-12 min hold 2% C. The columns were heated to 40 °C by a Dionex UltiMate 3000 Rapid Separations analytical column heater. The injection system was cleaned with an isopropanol-water solution (90:10, *v/v*), using the following settings: wash volume, 100 µL; wash speed, 4000 nL/s; loop wash factor, 2. The injection volume was 1 µL for every sample.

The HESI-II source conditions for data acquisition using full scan were adapted from previous works (Manier et al. 2019) and as follows: ionization mode, positive or negative; sheath gas, 60 AU; auxiliary gas, 10 AU; sweep gas, 3 AU; spray voltage, 3.50 kV in positive mode and −4.0 kV in negative mode; heater temperature, 320 °C; ion transfer capillary temperature, 320 °C; and S-lens RF level, 50.0. The settings for data acquisition in full scan were as follows: resolution, 140,000 fwhm; microscans, 1; automatic gain control (AGC) target, 5 × 105; maximum injection time, 200 ms; scan

range, m/z 50–750; polarity, negative or positive; spectrum data type, centroid. Significant features were identified using parallel reaction monitoring (PRM).

MoInt were additionally identified using parallel reaction monitoring (PRM), adapted from previous works, too (Manier et al. 2020). The parameters were: resolution, 70,000 fwhm; microscans, 1; AGC target, 5 × 105; maximum injection time, 200 ms; isolation window, 0.4 m/z; collision energy (CE), 10 eV, 20 eV, or 40 eV; spectrum data type, centroid. The inclusion list contained the monoisotopic masses of all successfully detected MoInt and a time window of their retention time ±30 s.

To equilibrate the system, at least two injections of pure methanol (for reversed phase chromatography) or eluent D (for normal phase chromatography) were performed prior to each experiment, followed by one injection of the respective pooled QC sample. The order of the samples was randomized, and for every five samples, the pooled QC sample was measured again to detect and correct batch effects as described by Wehrens et al. (Wehrens et al. 2016). Xcalibur Version 4.1.31.9 (TF, Dreieich, Germany) was used for data handling.

Table S1. Used internal standards, peak picking success on reversed-phase (RP) and hydrophilic interaction (HILIC) column, mass to charge ratio (*m/z*) and retention time (rt) for positive (pos) and negative (neg) ionization.

		peak picking							
		RP		HILIC		*m/z*		rt [s]	
spike solution	compound name	pos	neg	pos	neg	pos	neg	RP	HILIC
1	Glucose-d_7	×	✓	×	×	188.115	186.100	23	480
1	Palmitic acid-d_{31}	×	✓	×	✓	288.442	286.428	524	82
1	Tryptophan-d_5	✓	✓	✓	×	210.129	208.114	135	454
2	Glibenclamid	✓	✓	✓	✓	494.151	492.137	429	96
2	Trimipramine-d_3	✓	×	✓	×	298.236	296.221	347	295
3	Bisoprolol-d_5	✓	×	✓	×	331.264	329.249	274	326
3	Furosemide	×	✓	×	×	331.015	329.000	318	233
3	Hydrochlorothiazide	×	✓	×	✓	297.972	295.957	166	146
3	Telmisartan-d_7	✓	×	✓	×	522.288	520.274	373	170

Table S2. Peak picking parameters for the centwave algorithm, optimized for RP and HILIC column and positive (pos) and negative (neg) ionization. Ppm = allowed ppm deviation of mass traces, s/n threshold = signal-to-noise threshold, m/z diff. = minimum difference in m/z between to separated peaks.

Column	RP		HILIC	
Ionization	pos	neg	pos	neg
minimum peakwidth [s]	7.8	7.8	7.8	7.8
maximum peakwidth [s]	85	85	85	83
ppm	2.3	2.3	2.5	2.5
s/n treshold	13	13	11	28
m/z diff.	0.008	0.008	0.058	0.006
bandwith	0.2	0.2	0.7	0.8
Prefilter 1, scan number	7	7	7	12
Prefilter 2, scan abundance	100	100	100	2600

Table S3. Significance of differences between peak areas of IS, compared to extraction I, calculated using Welch´s *t*-Test. * = $p < 0.05$; ** = $p < 0.01$; *** = $p < 0.001$; **** = $p < 0.0001$, ns = not significant. n= 5 for each sample group. Raw data acquired on Phenyl-Hexyl column using positive ionization.

	Welch´s *t*-Test Significance of IS Peak Areas				
Compound Name	Extraction II	Extraction III	Extraction IV	Extraction III_B	Extraction IV_B
Bisoprolol-d_5	ns	*	ns	ns	ns
Glibenclamide	ns	ns	ns	**	*
Telmisartan-d_7	ns	ns	ns	ns	ns
Trimipramine-d_3	**	**	ns	****	***
Tryptophan-d_5	ns	ns	**	ns	*

Table S4. Significance of differences between peak areas of IS, compared to extraction I, calculated using Welch´s *t*-Test. * = $p < 0.05$; ** = $p < 0.01$; *** = $p < 0.001$; **** = $p < 0.0001$, ns = not significant. n= 5 for each sample group. Raw data acquired on Phenyl-Hexyl column using negative ionization.

	Welch´s *t*-Test Significance of IS Peak Areas				
Compound Name	Extraction II	Extraction III	Extraction IV	Extraction III_B	Extraction IV_B
Furosemide	ns	ns	ns	**	*
Glibenclamide	ns	ns	ns	ns	ns
Glucose-d_7	*	ns	ns	ns	**
Hydrochlorothiazide	*	ns	ns	ns	ns
Palmitic acid-d_{31}	ns	***	***	***	***
Tryptophan-d_5	ns	ns	ns	ns	**

Table S5. Significance of differences between peak areas of IS, compared to extraction I, calculated using Welch´s *t*-Test. * = $p < 0.05$; ** = $p < 0.01$; *** = $p < 0.001$; **** = $p < 0.0001$, ns = not significant. n= 5 for each sample group. Raw data acquired on HILIC column using positive ionization.

	Welch´s *t*-Test Significance of IS Peak Areas				
Compound Name	Extraction II	Extraction III	Extraction IV	Extraction III_B	Extraction IV_B
Bisoprolol-d_5	*	ns	*	*	ns
Glibenclamide	ns	**	**	**	**
Telmisartan-d_7	*	*	**	*	*
Trimipramine-d_3	*	*	**	****	**
Tryptophan-d_5	*	ns	**	*	**

Table S6. Significance of differences between peak areas of IS, compared to extraction I, calculated using Welch´s *t*-Test. * = $p < 0.05$; ** = $p < 0.01$; *** = $p < 0.001$; **** = $p < 0.0001$, ns = not significant. n= 5 for each sample group. Raw data acquired on HILIC column using negative ionization.

	Welch´s *t*-Test Significance of IS Peak Areas				
Compound Name	Extraction II	Extraction III	Extraction IV	Extraction III_B	Extraction IV_B
Glibenclamide	ns	*	*	ns	**
Hydrochlorothiazide	ns	ns	ns	*	*
Palmitic acid-d_{31}	ns	***	***	***	***

Table S7. Molecules of Interest (MoInt) selected based on literature (Chai, Cui et al. 2016, Vrieling, Alisjahbana et al. 2019, Albors-Vaquer, Rizvi et al. 2020, Ding, Raterink et al. 2020, Conde, Laires et al. 2021) and preliminary experiments for further evaluation, and the used m/z Values for analysis.

	m/z Value	
Compound Name	positive	negative
2-Aminobenzoic acid	138.0550	136.0404
2-Aminobutyrate	104.0706	102.0561
3-dehydroxycarnitine	146.1176	144.1030
3-Hydroxybutyrate	105.0546	103.0401
3-Ketocholesterol	385.3465	383.3319
3-Methoxytyrosine	212.0917	210.0772
5-hydroxylysine	163.1077	161.0932
5-hydroxytryptophane	221.0921	219.0775
5-Thymidylic acid	323.0639	321.0493
Adenosine	268.1040	266.0895
Alanine	90.0550	88.0404
Aminoadipic acid	162.0761	160.0615
Arginine	175.1190	173.1044
Asparagine	133.0608	131.0462
Aspartic acid	134.0448	132.0302
Choline	105.1143	103.0997
Citric acid	193.0343	191.0197
Citrulline	176.1030	174.0884
Creatine	132.0768	130.0622
Cysteine	122.0270	120.0125
Cytidine	244.0928	242.0782
Dimethylarginine	203.1503	201.1357
Ethanolamine	62.0600	60.0455
Glibenclamid	494.1511	492.1365
Glucose	181.0707	179.0561
Glutamic acid	148.0604	146.0459
Glutamine	147.0764	145.0619
Glycerophosphocholine	258.1101	256.0955
Histidine	156.0768	154.0622
Hydroxyproline	132.0655	130.0510

Table S7. Continued.

Compound Name	*m/z* Value	
	positive	negative
Hypoxanthine	137.0458	135.0312
Inosine	269.0880	267.0735
Inosinic acid	349.0544	347.0398
Lactic acid	91.0390	89.0244
Leucine	132.1019	130.0874
Lysine	147.1128	145.0983
Methionine	150.0583	148.0438
N6,N6,N6-trimethyl-l-lysine	189.1598	187.1452
Nicotinamide	123.0553	121.0407
N-Palmitoyl-D-Sphingosine	538.5194	536.5048
O-phosphoethanolamine	142.0264	140.0118
Ornithine	133.0972	131.0826
Palmityolethanolamide	300.2897	298.2752
Phenylalanine	166.0863	164.0717
Pipecolic acid	130.0863	128.0717
Proline	116.0706	114.0561
Propionylcarnitine	218.1387	216.1241
Propylene Glycol	77.0597	75.0452
Putrescine	89.1073	87.0928
Serine	106.0499	104.0353
Stearoylethanolamide	328.3210	326.3065
Threonine	120.0655	118.0510
Tryptophan	205.0972	203.0826
Tyrosine	182.0812	180.0662
Urea	61.0396	59.0251
Uridine	245.0768	243.0623
Valine	118.0863	116.0717
Xanthine	153.0407	151.0261
γ-Glutamylalanine	219.0975	217.0830

Table S8. Significance of differences between peak areas of MoInts, compared to extraction I, calculated using Welch´s *t*-Test. * = $p < 0.05$; ** = $p < 0.01$; *** = $p < 0.001$; **** = $p < 0.0001$, ns = not significant. n= 5 for each sample group. Raw data acquired on Phenyl-Hexyl column using positive ionization.

	Welch´s t-Test Significance of MoInt Peak Areas				
	Extraction Procedure				
Compound Name	II	III	IV	III_B	IV_B
2-Aminobenzoic acid	ns	ns	*	ns	**
3-Dehydroxycarnitine	ns	ns	*	*	**
3-Ketocholesterol	**	****	****	****	****
3-Methoxytyrosine	*	ns	*	*	****
Adenosine	*	***	****	**	**
Aminoadipic acid	ns	ns	ns	ns	ns
Arginine	ns	****	***	****	***
Asparagine	ns	ns	**	ns	*
Aspartic acid	ns	ns	ns	ns	ns
Creatine	*	ns	ns	*	***
Cytidine	ns	*	**	**	***
Glutamic acid	ns	ns	ns	ns	ns
Glutamine	ns	ns	ns	***	***
Glycerophosphocholine	ns	**	**	***	***
Histidine	ns	****	***	**	ns
Hypoxanthine	***	***	***	ns	*
Inosine	ns	****	****	***	****
Inosinic acid	ns	**	****	***	****
Leucine	**	ns	*	*	ns
Lysine	ns	****	***	****	***
Methionine	ns	**	**	**	*
N6,N6,N6-trimethyl-l-lysine	ns	ns	ns	**	ns
Nicotinamide	**	*	ns	ns	**
N-Palmitoyl-D-Sphingosine	***	***	***	***	***
Phenylalanine	*	****	***	***	***
Pipecolic acid	ns	ns	ns	ns	ns
Propionylcarnitine	ns	ns	ns	ns	ns
Stearoylethanolamide	ns	ns	ns	ns	ns
Threonine	*	***	****	*	*
Tryptophan	ns	**	**	**	ns
Tyrosine	**	ns	ns	*	ns
Uridine	ns	ns	ns	ns	ns
Xanthine	ns	****	***	***	**

Table S9. Significance of differences between peak areas of MoInts, compared to extraction I, calculated using Welch´s *t*-Test. * = $p < 0.05$; ** = $p < 0.01$; *** = $p < 0.001$; **** = $p < 0.0001$, ns = not significant. n= 5 for each sample group. Raw data acquired on Phenyl-Hexyl column using negative ionization.

	Welch´s t-Test Significance of MoInt Peak Areas				
	Extraction Procedure				
Compound Name	II	III	IV	III_B	IV_B
Glucose	ns	*	**	ns	****
Glutamic acid	*	ns	ns	ns	***
Glutamine	*	ns	ns	ns	****
Histidine	ns	*	ns	*	*
Hypoxanthine	**	*	***	**	ns
Inosine	ns	****	****	***	***
Inosinic acid	ns	*	****	****	****
Methionine	ns	*	***	****	**
Phenylalanine	ns	**	**	***	**
Tryptophan	ns	**	*	**	ns
Tyrosine	ns	**	ns	**	ns
Xanthine	ns	**	***	****	***

Table S10. Significance of differences between peak areas of MoInts, compared to extraction I, calculated using Welch´s t-Test. * = $p < 0.05$; ** = $p < 0.01$; *** = $p < 0.001$; **** = $p < 0.0001$, ns = not significant. n= 5 for each sample group. Raw data acquired on HILIC column using positive ionization.

	Welch´s t-Test Significance of MoInt Peak Areas				
	Extraction Procedure				
Compound Name	II	III	IV	III_B	IV_B
2-Aminobenzoic acid	ns	ns	ns	ns	**
Adenosine	ns	*	****	*	ns
Arginine	ns	**	*	****	**
Creatine	ns	ns	ns	ns	**
Cytidine	*	**	**	**	**
Dimethylarginine	*	****	**	***	*
Glutamic acid	ns	ns	ns	*	ns
Glutamine	*	ns	ns	ns	*
Glycerophosphocholine	ns	ns	**	**	**
Hypoxanthine	ns	**	**	*	ns
Inosine	ns	****	****	****	***
Inosinic acid	ns	**	****	****	****
Leucine	**	ns	**	ns	ns
Lysine	ns	***	ns	****	**
Methionine	**	*	*	*	ns
Propionylcarnitine	ns	ns	**	**	****
Tryptophan	**	ns	ns	ns	ns
Tyrosine	*	ns	ns	ns	ns
Xanthine	*	***	***	***	**

Table S11. Significance of differences between peak areas of MoInts, compared to extraction I, calculated using Welch´s *t*-Test. * = $p < 0.05$; ** = $p < 0.01$; *** = $p < 0.001$; **** = $p < 0.0001$, ns = not significant. n= 5 for each sample group. Raw data acquired on HILIC column using negative ionization.

	Welch´s *t*-Test Significance of MoInt Peak Areas				
Compound Name	Extraction II	Extraction III	Extraction IV	Extraction III_B	Extraction IV_B
Aminoadipic acid	ns	ns	*	ns	***
Arginine	ns	****	**	***	**
Cytidine	ns	**	**	***	*
Glutamine	ns	ns	ns	**	**
Hypoxanthine	***	**	*	*	*
Inosine	ns	****	****	***	***
Inosinic acid	ns	**	**	**	***
Methionine	*	ns	ns	ns	*
Phenylalanine	***	****	**	**	ns
Tyrosine	**	ns	ns	ns	ns
Uridine	ns	*	ns	****	****
Xanthine	ns	***	***	***	**

Table S12. Results of the pathway analysis of all detected MoInt, using Metaboanalyst version 5.0. The 33 detected pathways, the amount of total metabolites in each pathways, the expected amount of correlations (hits), the amount of correlated MoInts, the raw p-values and the impact value from pathway topology analysis are presented.

		Hits			
Pathway	Metabolites	expected	MoInt	p-Value	Impact
Aminoacyl-tRNA biosynthesis	48	1	13	9.19E-13	0.00
Lysine degradation	25	0.52	5	1.11E-04	0.13
Arginine biosynthesis	14	0.29	4	1.34E-04	0.19
Purine metabolism	66	1.38	6	1.86E-03	0.18
Alanine, aspartate and glutamate metabolism	27	0.56	4	1.94E-03	0.58
Phenylalanine, tyrosine and tryptophan biosynthesis	4	0.08	2	2.46E-03	1.00
Histidine metabolism	15	0.31	3	3.16E-03	0.22
Nitrogen metabolism	6	0.13	2	6.00E-03	0.00
D-Glutamine and D-glutamate metabolism	6	0.13	2	6.00E-03	0.50
Valine, leucine and isoleucine biosynthesis	8	0.17	2	1.09E-02	0.00
Phenylalanine metabolism	8	0.17	2	1.09E-02	0.36
Nicotinate and nicotinamide metabolism	14	0.29	2	3.28E-02	0.22
Arginine and proline metabolism	38	0.79	3	4.24E-02	0.15
Pyrimidine metabolism	41	0.86	3	5.13E-02	0.02
beta-Alanine metabolism	18	0.38	2	5.24E-02	0.00
Glyoxylate and dicarboxylate metabolism	32	0.67	2	1.42E-01	0.00
Glycine, serine and threonine metabolism	33	0.69	2	1.49E-01	0.02
Ubiquinone and other terpenoid-quinone biosynthesis	9	0.19	1	1.73E-01	0.00
Biotin metabolism	10	0.21	1	1.91E-01	0.00
Tryptophan metabolism	39	0.81	2	1.95E-01	0.14
Butanoate metabolism	15	0.31	1	2.72E-01	0.00
Fructose and mannose metabolism	20	0.42	1	3.46E-01	0.00
Ether lipid metabolism	20	0.42	1	3.46E-01	0.00
Sphingolipid metabolism	21	0.44	1	3.60E-01	0.27
Glycolysis / Gluconeogenesis	26	0.54	1	4.25E-01	0.00
Galactose metabolism	27	0.56	1	4.37E-01	0.03
Glutathione metabolism	28	0.58	1	4.49E-01	0.02
Porphyrin and chlorophyll metabolism	30	0.63	1	4.72E-01	0.00
Cysteine and methionine metabolism	33	0.69	1	5.05E-01	0.10
Glycerophospholipid metabolism	38	0.79	1	5.56E-01	0.04
Amino sugar and nucleotide sugar metabolism	39	0.81	1	5.66E-01	0.00
Valine, leucine and isoleucine degradation	40	0.84	1	5.75E-01	0.00
Tyrosine metabolism	42	0.88	1	5.93E-01	0.14

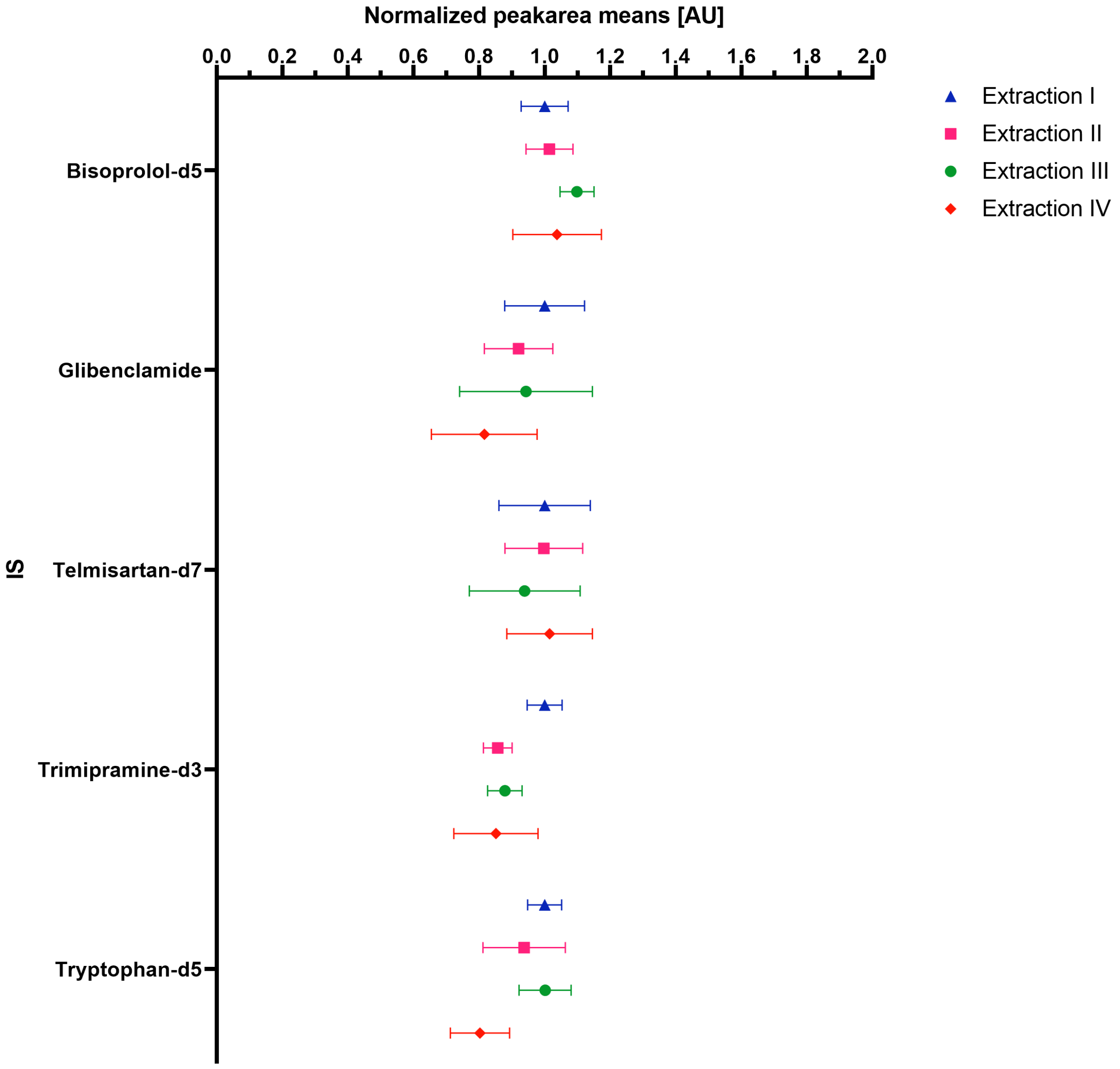

Figure S2. Normalized mean peak areas and standard deviation of internal standard after analysis using the Phenyl-Hexyl column and positive ionization. n= 5 for each sample group.

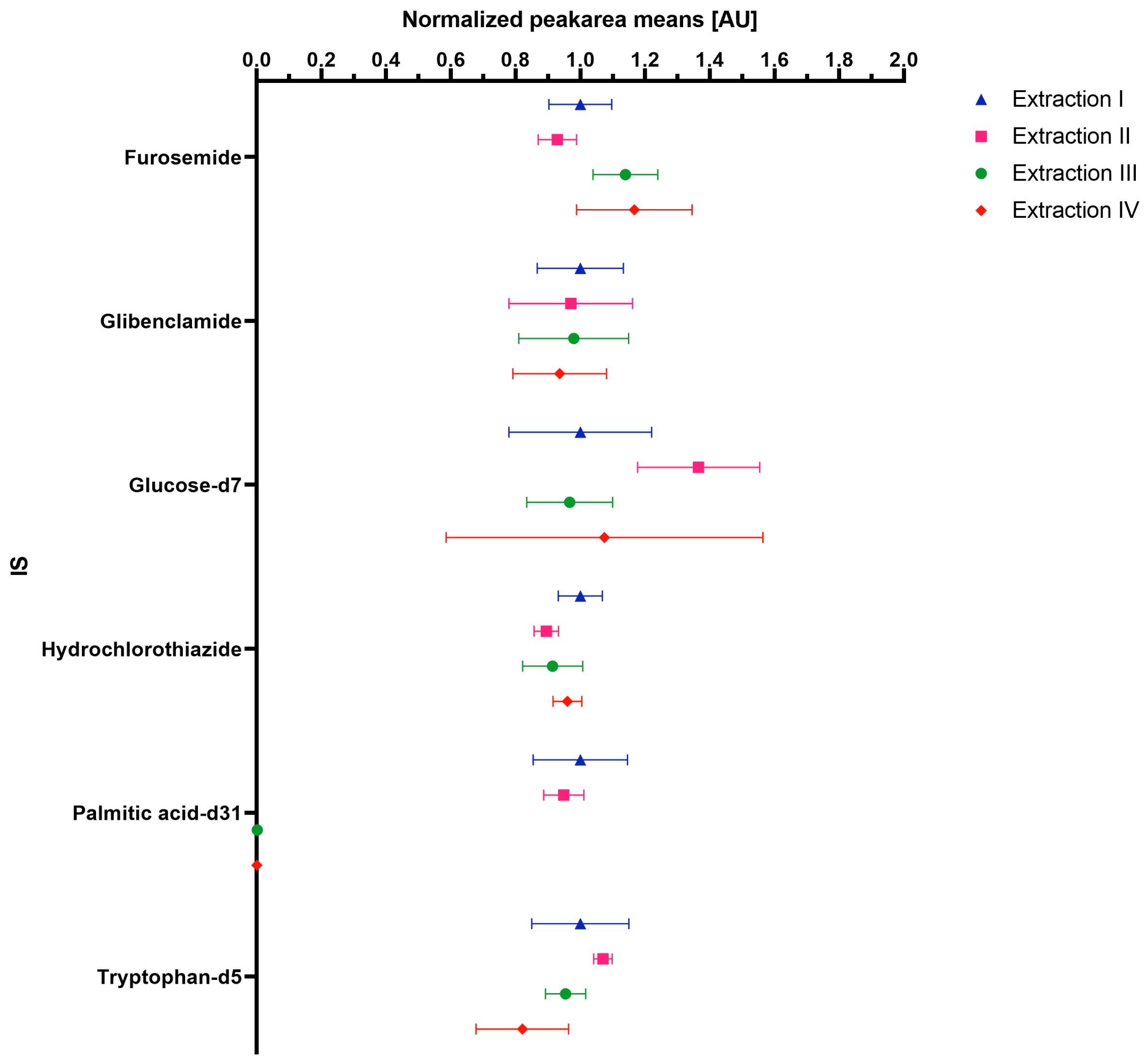

Figure S3. Normalized mean peak areas and standard deviation of each internal standard after analysis using the Phenyl-Hexyl column and negative ionization. n= 5 for each sample group.

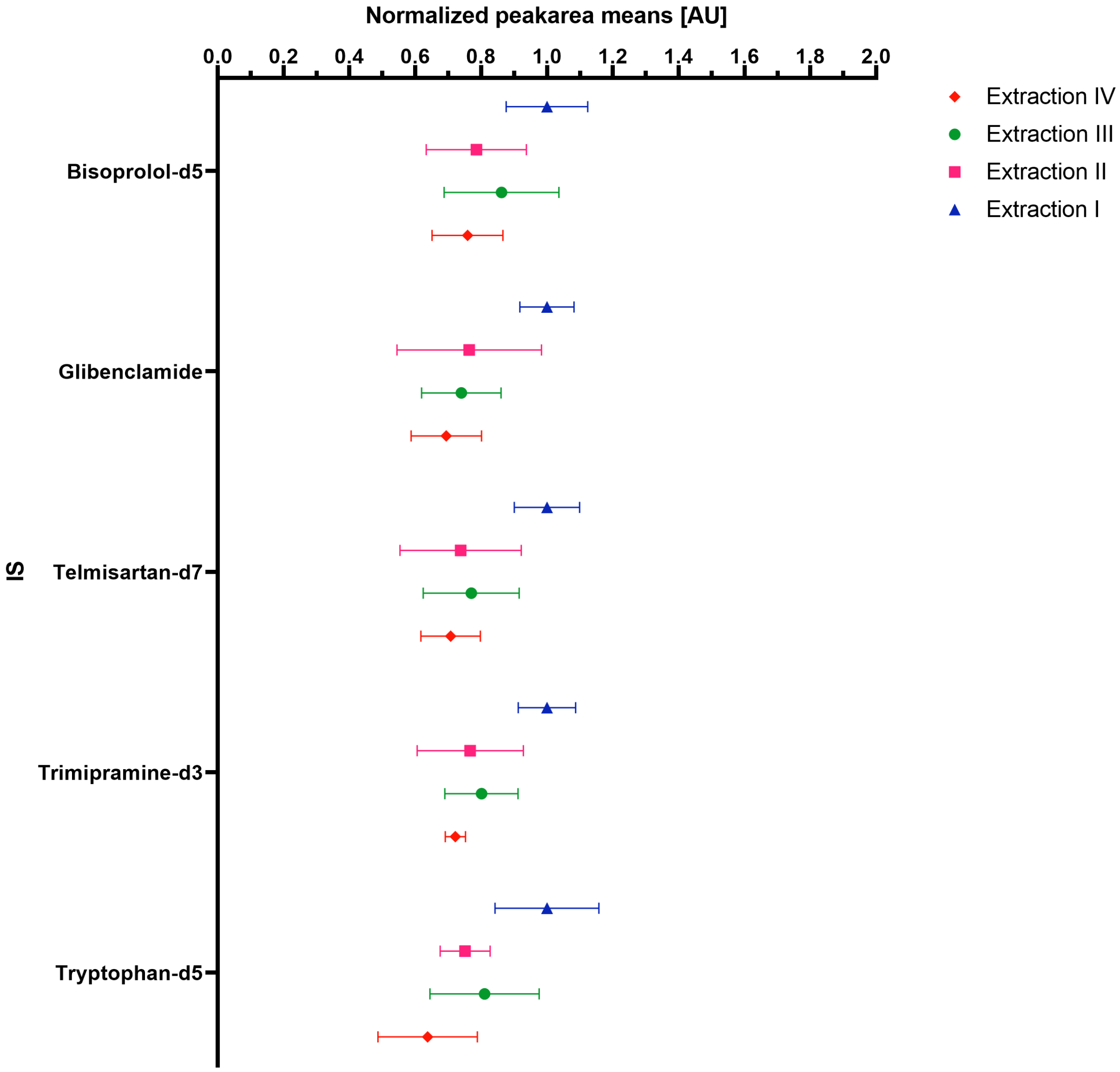

Figure S4. Normalized mean peak areas and standard deviation of each internal standard after analysis using the HILIC column and positive ionization. n= 5 for each sample group.

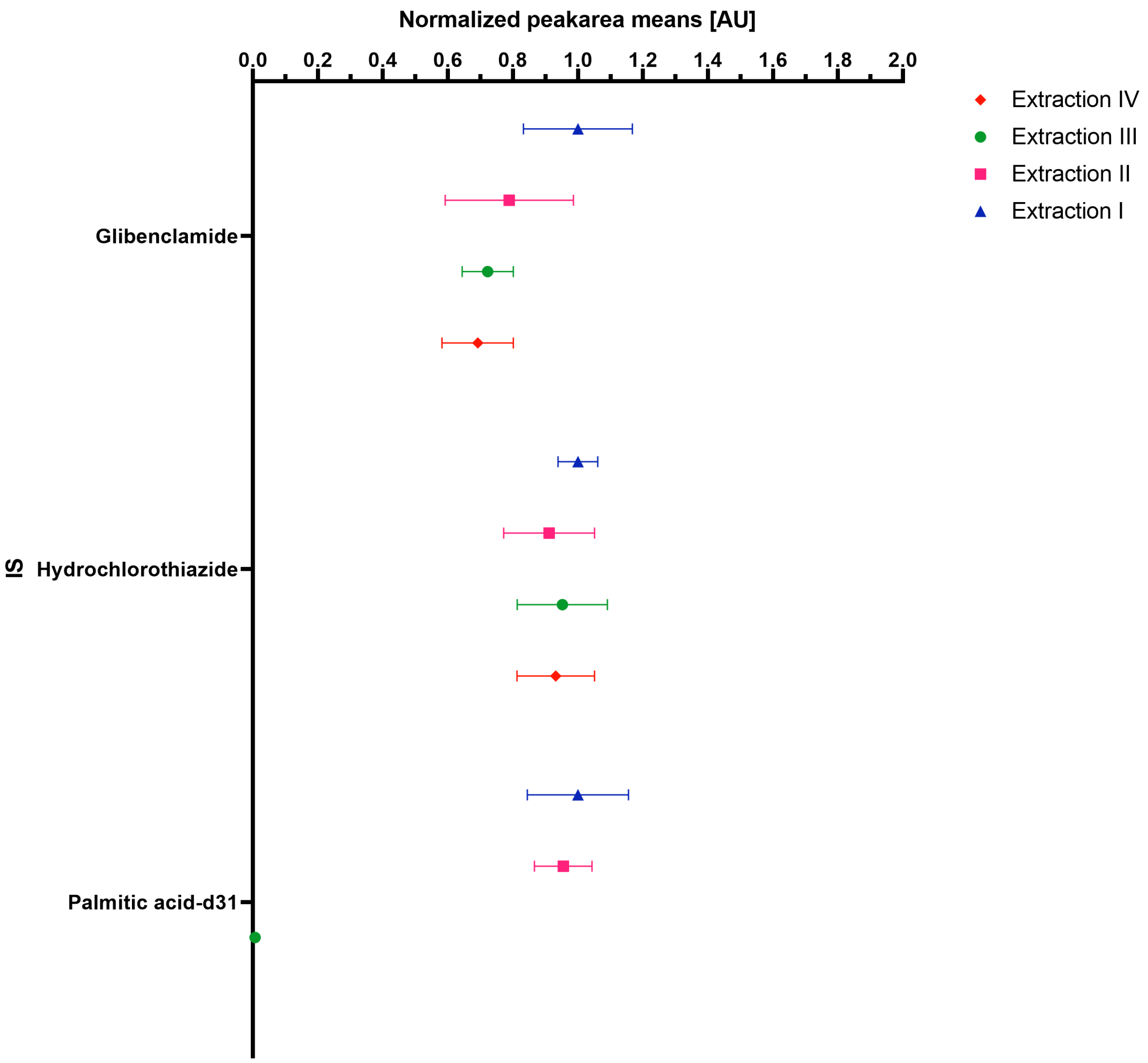

Figure S5. Normalized mean peak areas and standard deviation of each internal standard after analysis using the HILIC column and negative ionization. n= 5 for each sample group.

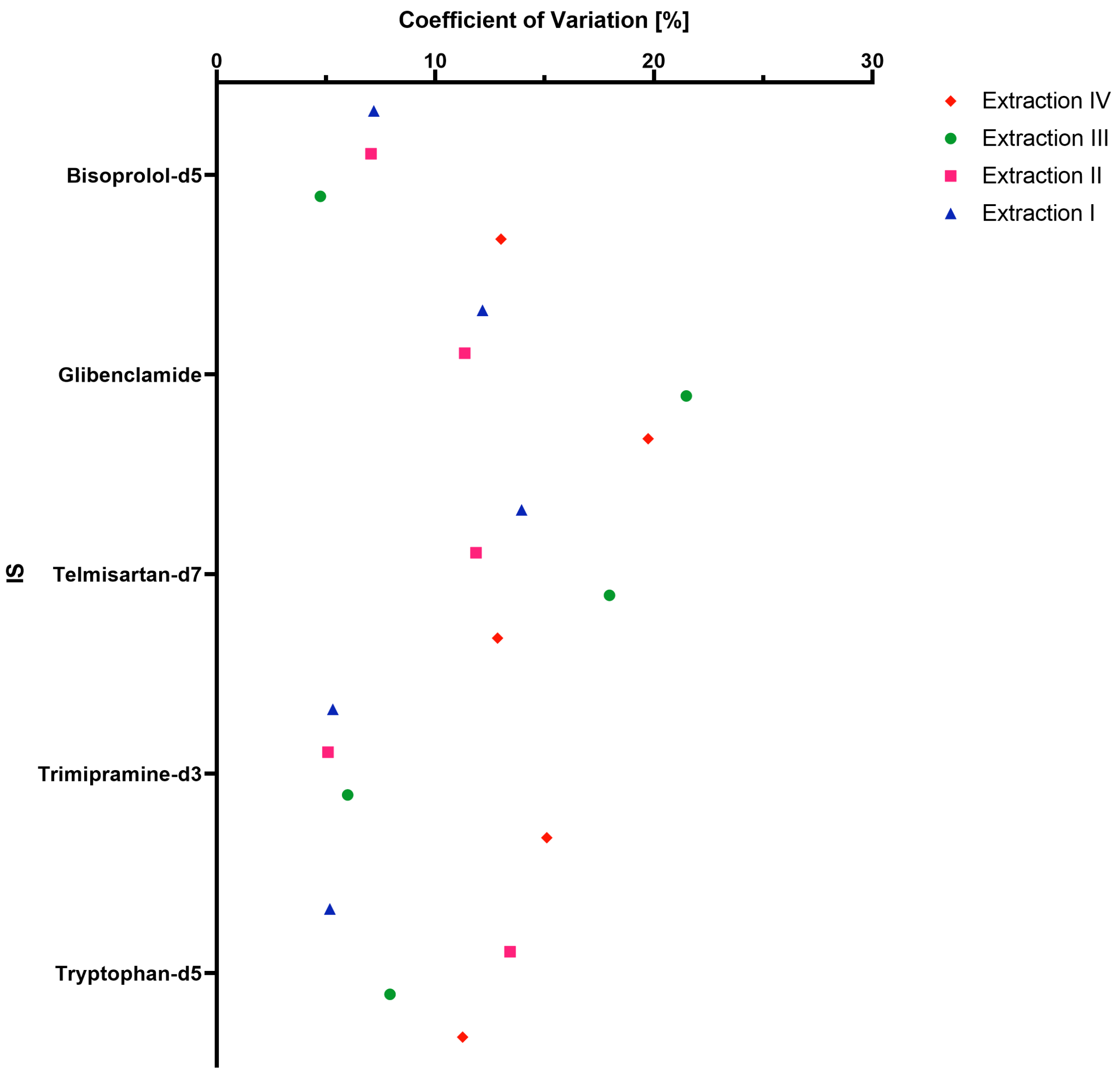

Figure S6. Coefficient of variation of each internal standard after analysis using the Phenyl-Hexyl column and positive ionization. n= 5 for each sample group.

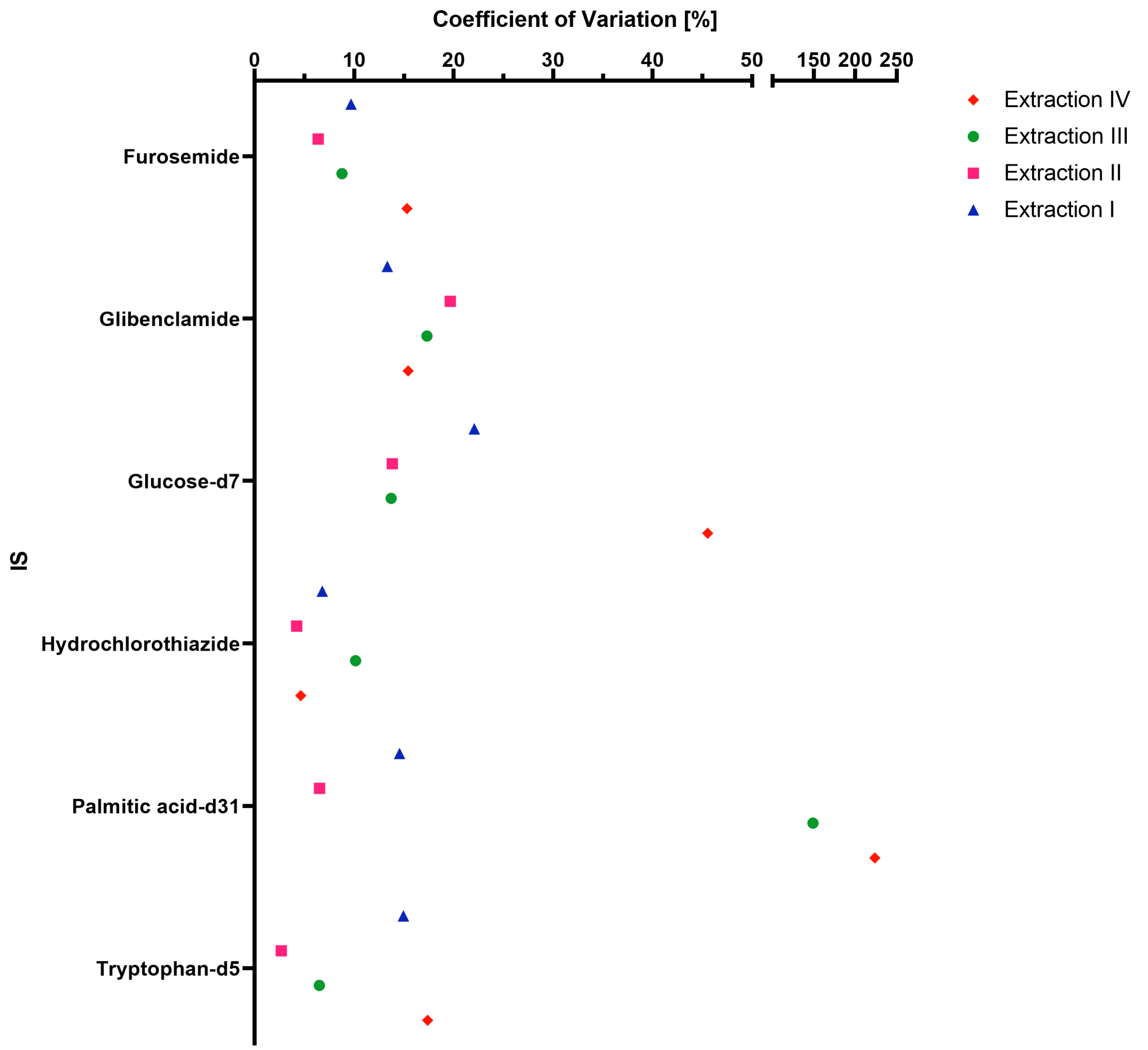

Figure S7. Coefficient of variation of each internal standard after analysis using the Phenyl-Hexyl column and negative ionization. n= 5 for each sample group.

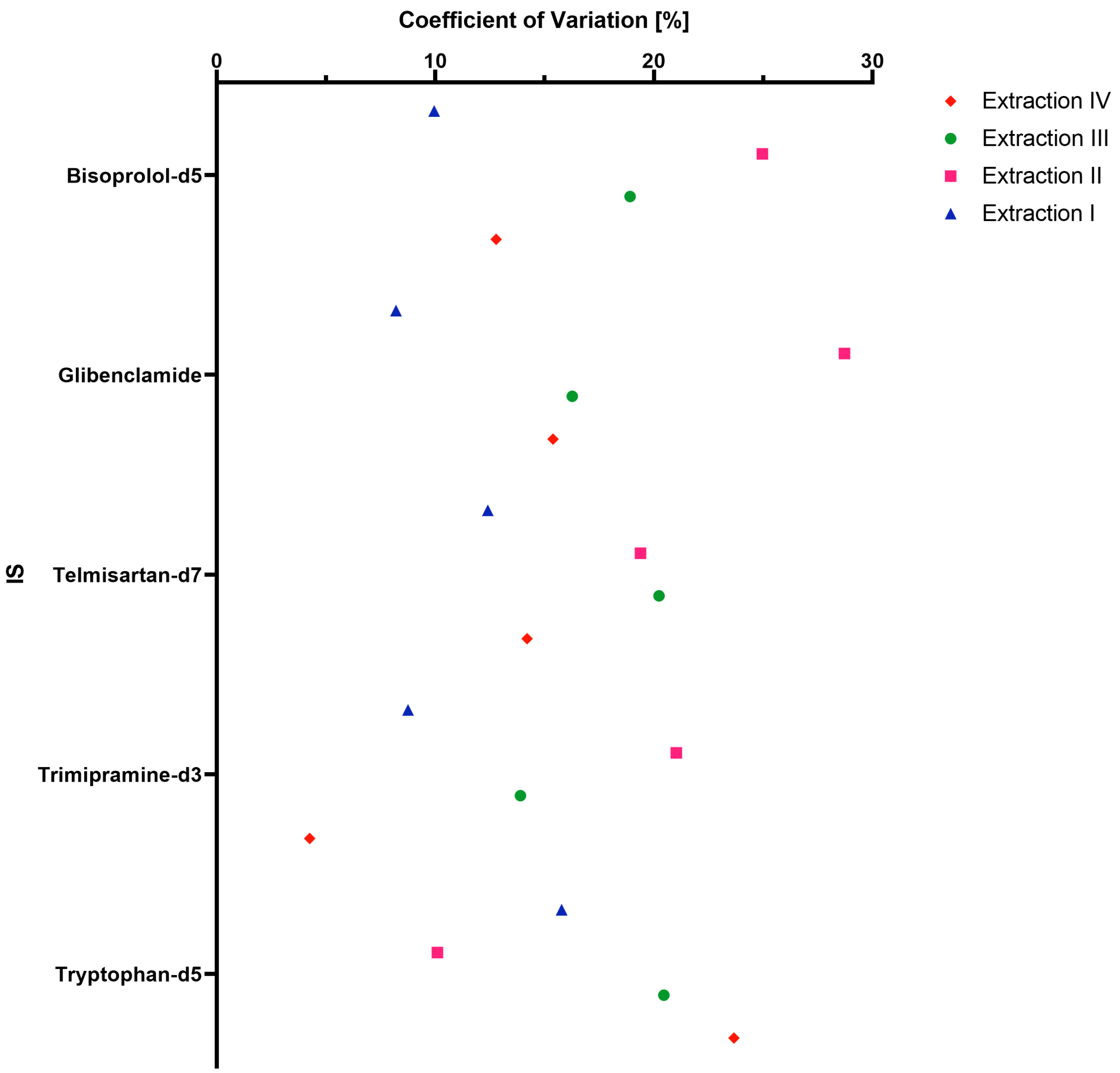

Figure S8. Coefficient of variation of each internal standard after analysis using the HILIC column and positive ionization. n= 5 for each sample group.

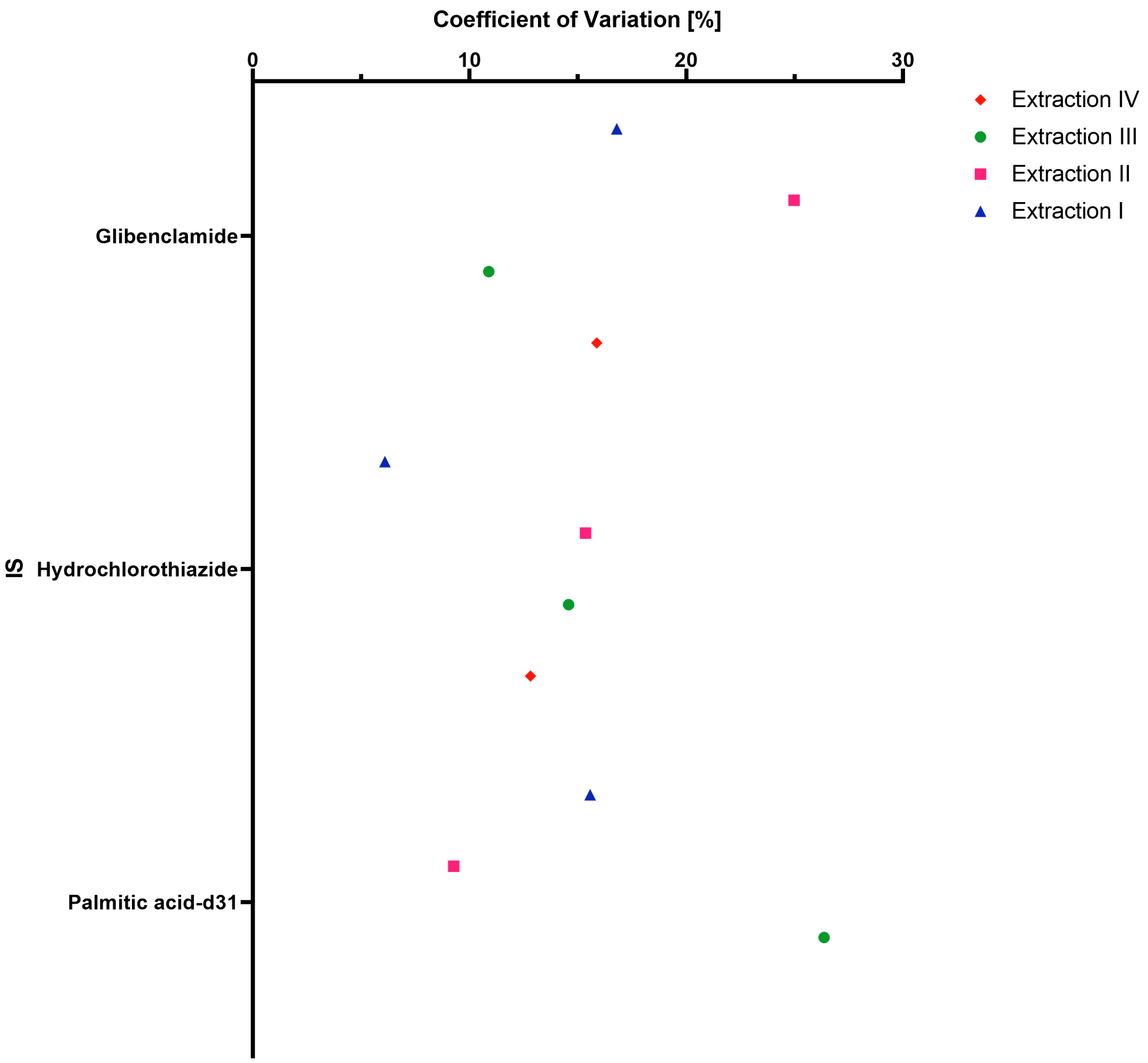

Figure S9. Coefficient of variation of each internal standard after analysis using the HILIC column and negative ionization. n= 5 for each sample group.

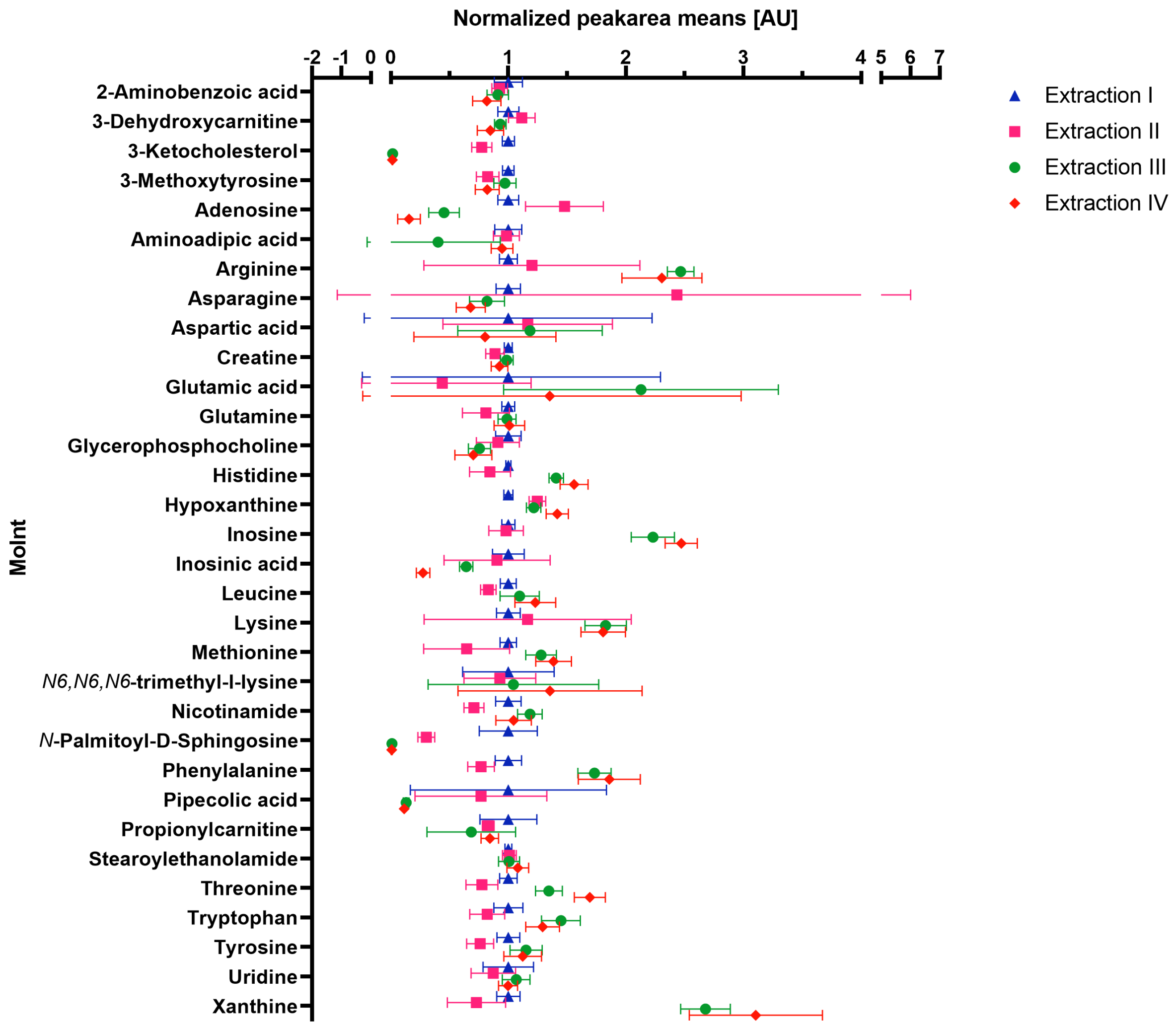

Figure S10. Normalized mean peak areas and standard deviation of each molecule of interest after analysis using the Phenyl-Hexyl column and positive ionization. n= 5 for each sample group. MoInt not detected in individual samples were excluded for 3-Ketocholesterol and *N*-Palmitoyl-D-Sphingosine.

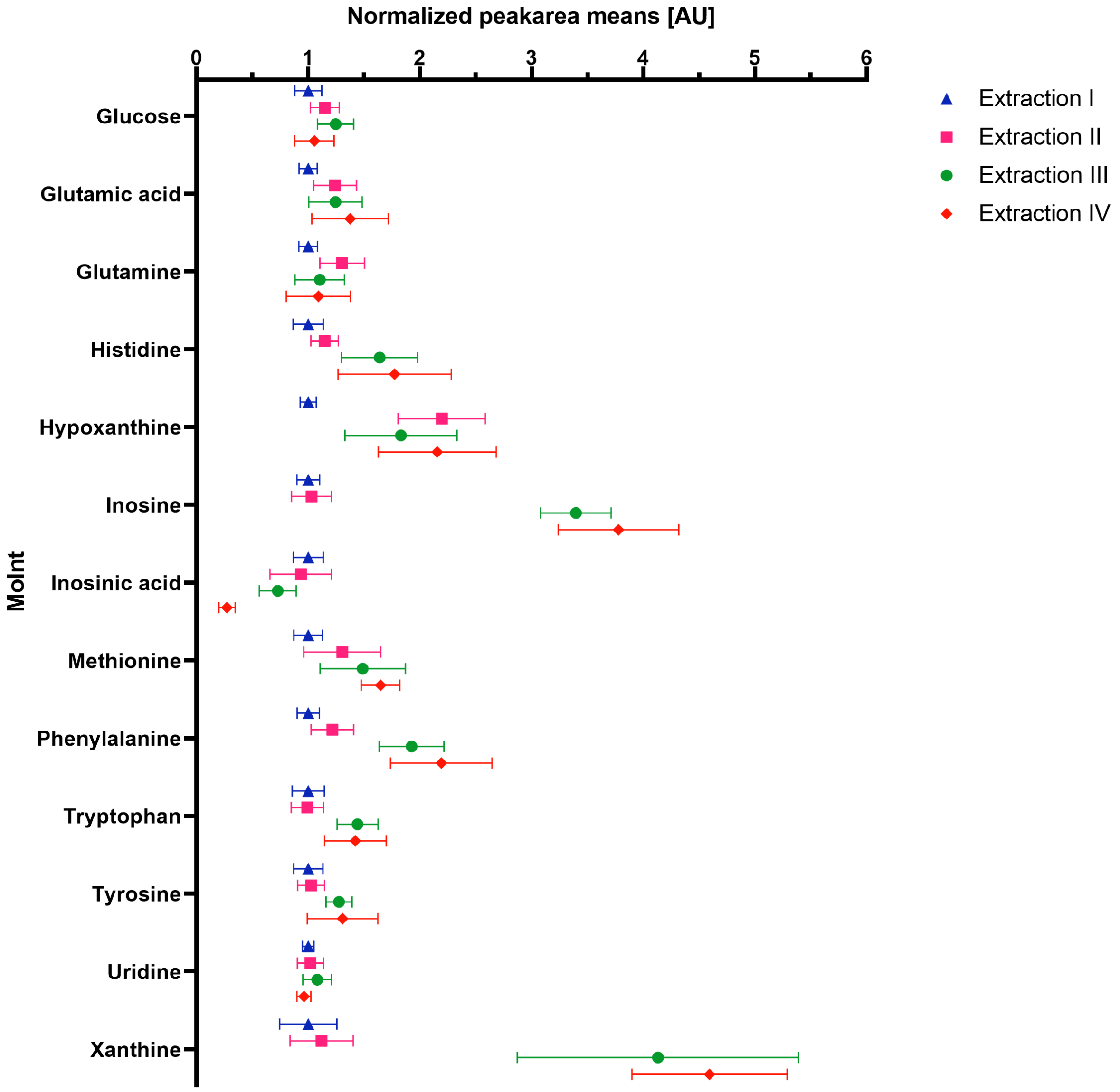

Figure S11. Normalized mean peak areas and standard deviation of each molecule of interest after analysis using the Phenyl-Hexyl column and negative ionization. n= 5 for each sample group.

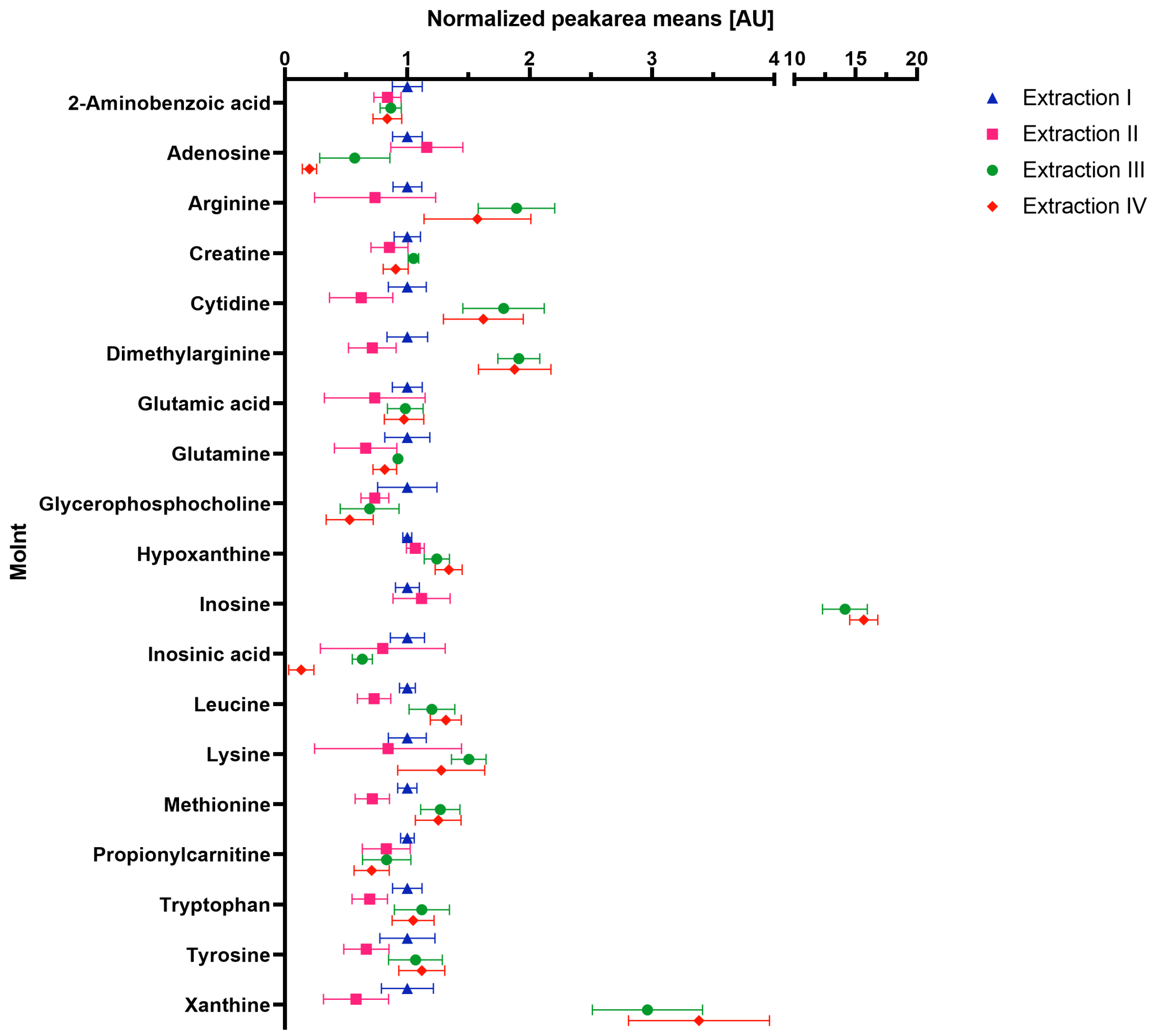

Figure S12. Normalized mean peak areas of each molecule of interest after analysis using the HILIC column and positive ionization. n= 5 for each sample group. MoInt not detected in individual samples were excluded for Inosinic acid and *N*-Palmitoyl-D-Sphingosine.

.

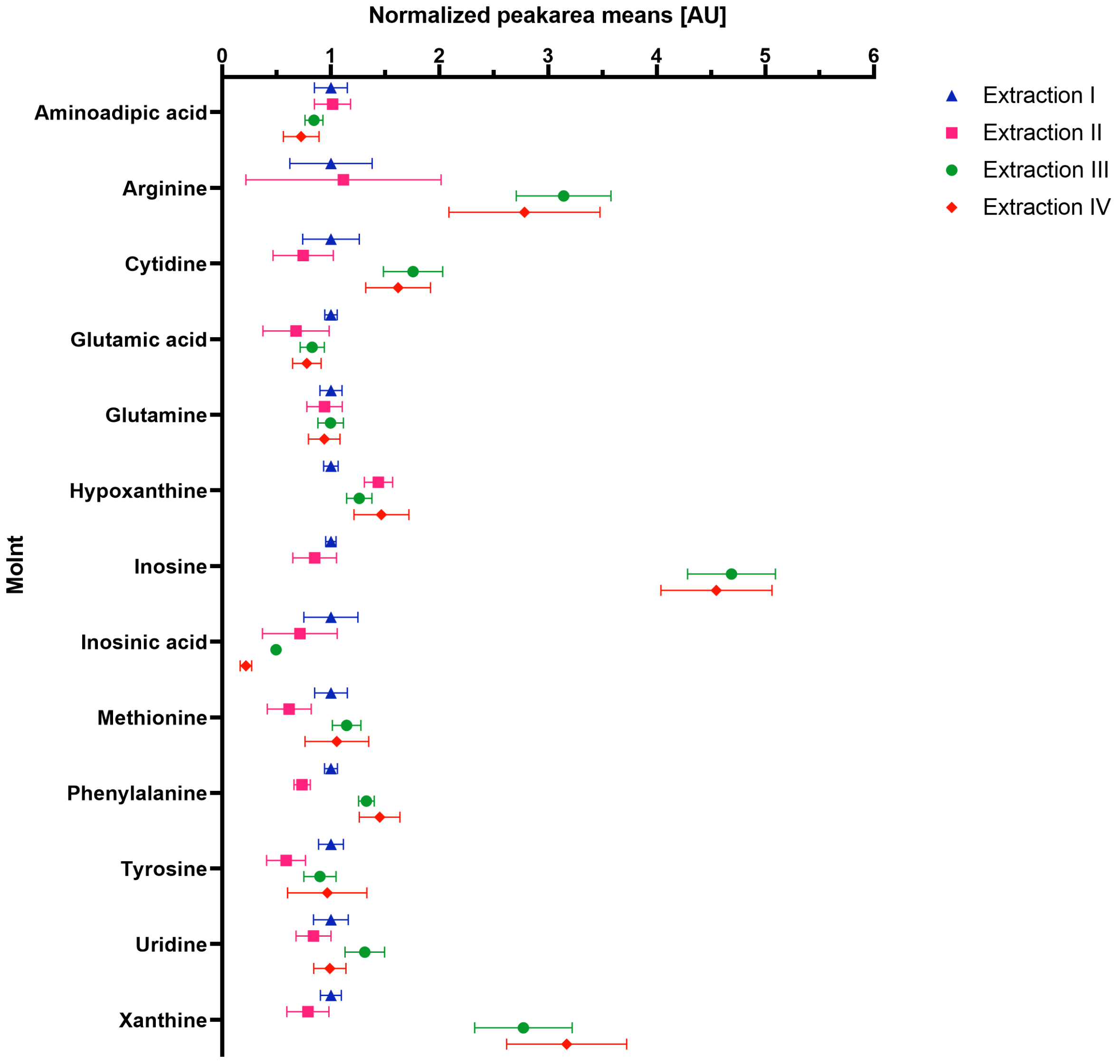

Figure S13. Normalized mean peak areas of each molecule of interest after analysis using the HILIC column and negative ionization. n= 5 for each sample group.

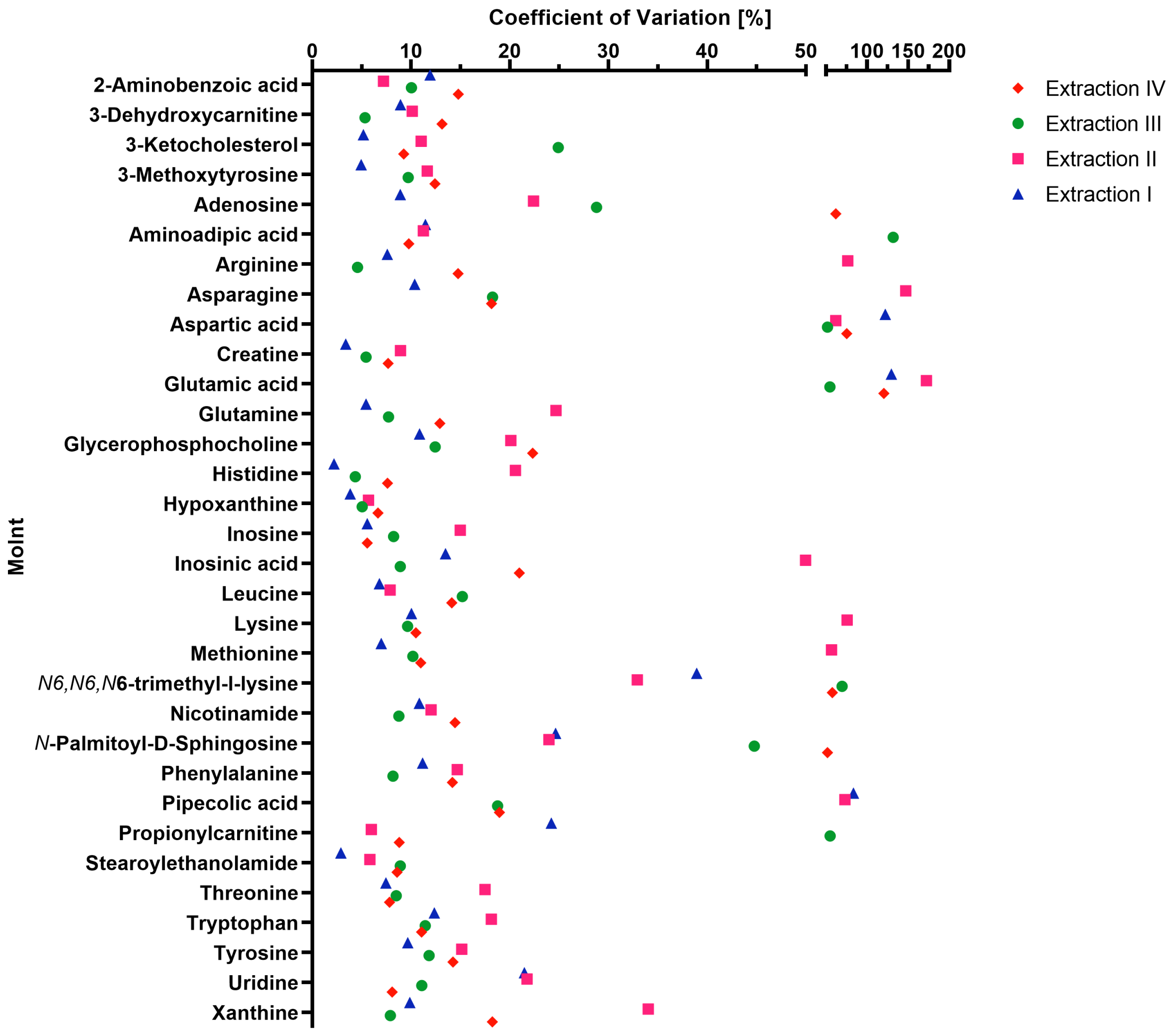

Figure S14. Coefficient of variation of each molecule of interest after analysis using the Phenyl-Hexyl column and positive ionization. n= 5 for each sample group. MoInt not detected in individual samples were excluded for 3-Ketocholesterol and *N*-Palmitoyl-D-Sphingosine.

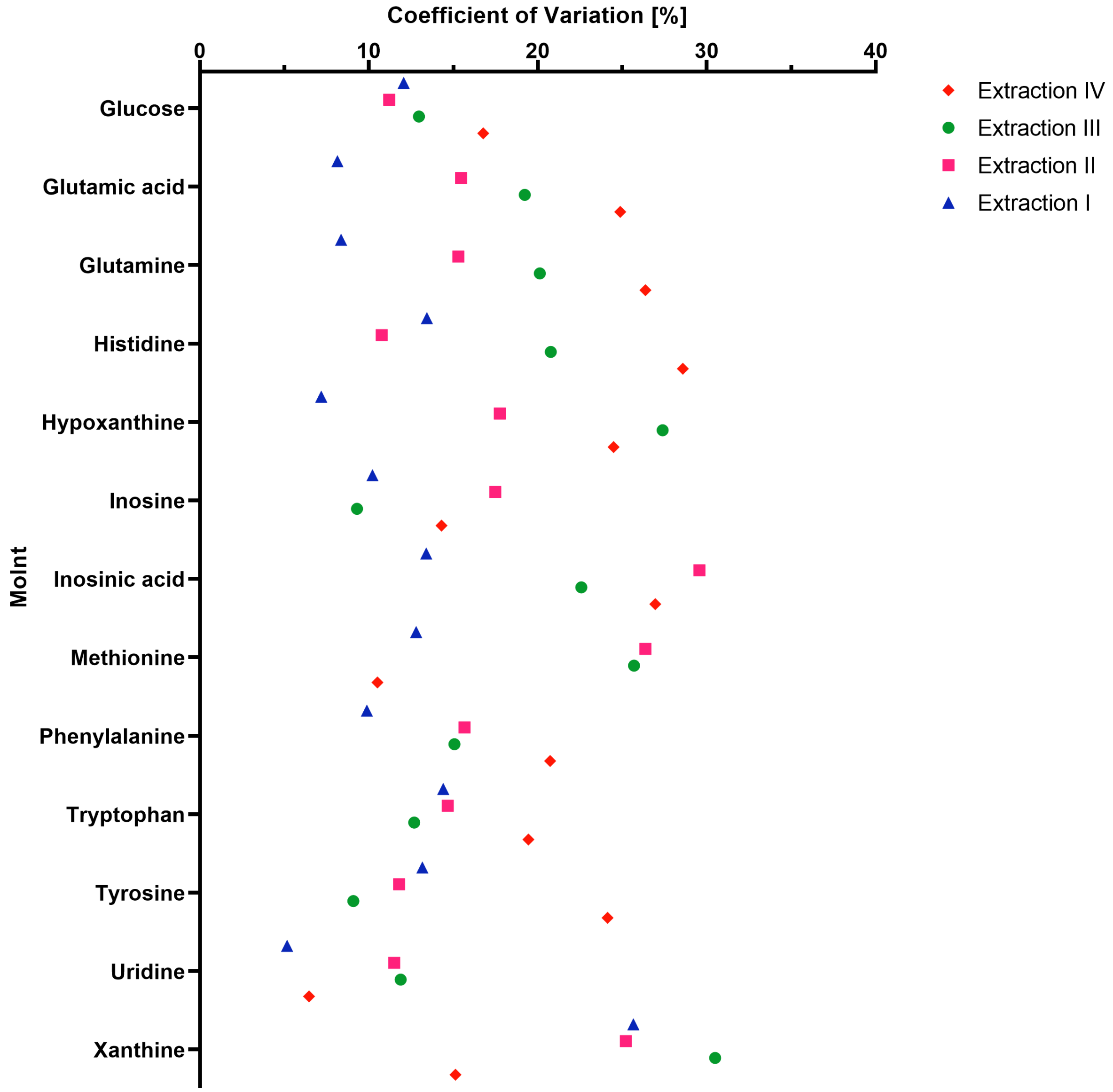

Figure S15. Coefficient of variation of each molecule of interest after analysis using the Phenyl-Hexyl column and negative ionization. n= 5 for each sample group.

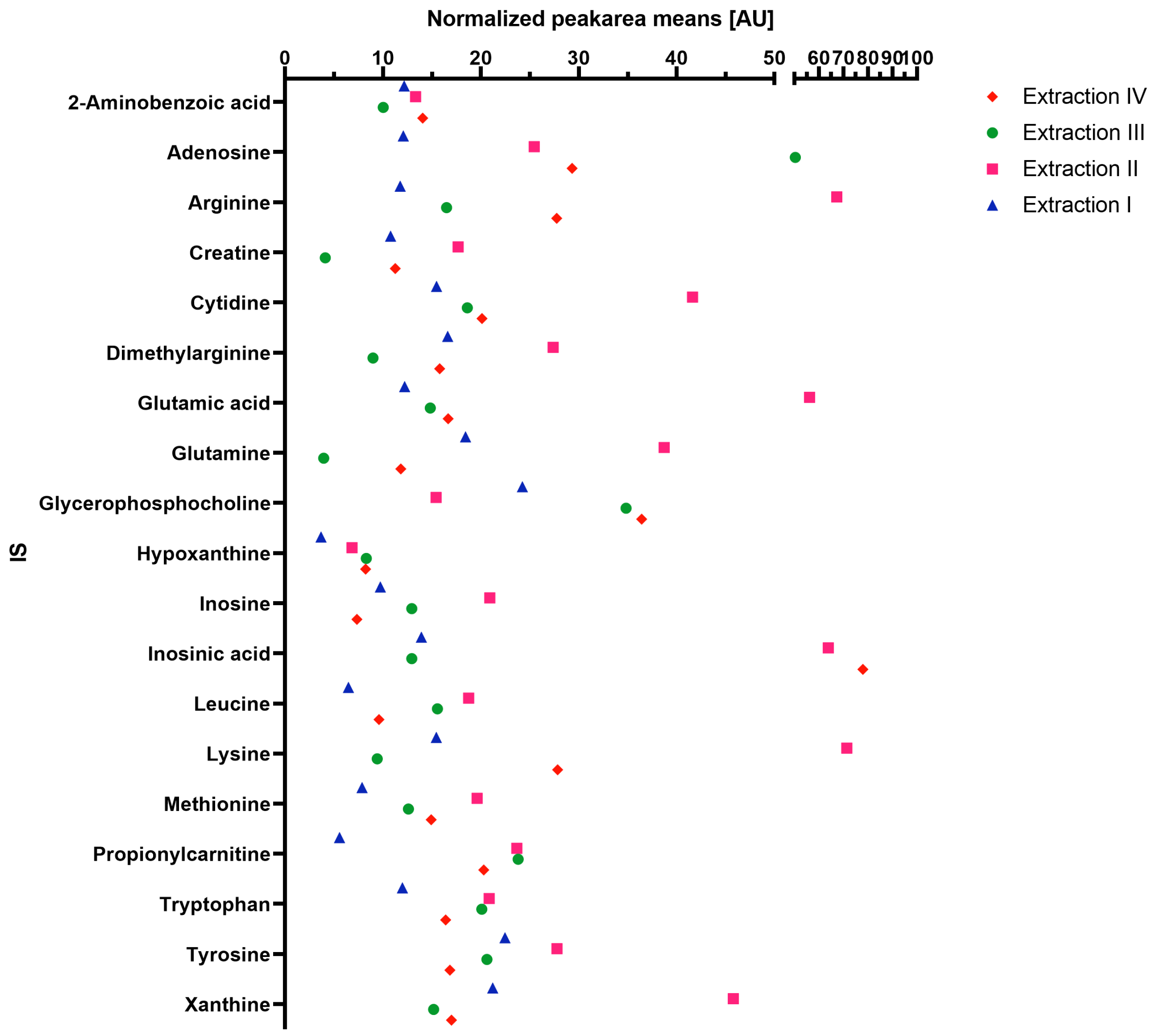

Figure S16. Coefficient of variation of each molecule of interest after analysis using the HILIC column and positive ionization. n= 5 for each sample group. MoInt not detected in individual samples were excluded for Inosinic acid and *N*-Palmitoyl-D-Sphingosine.

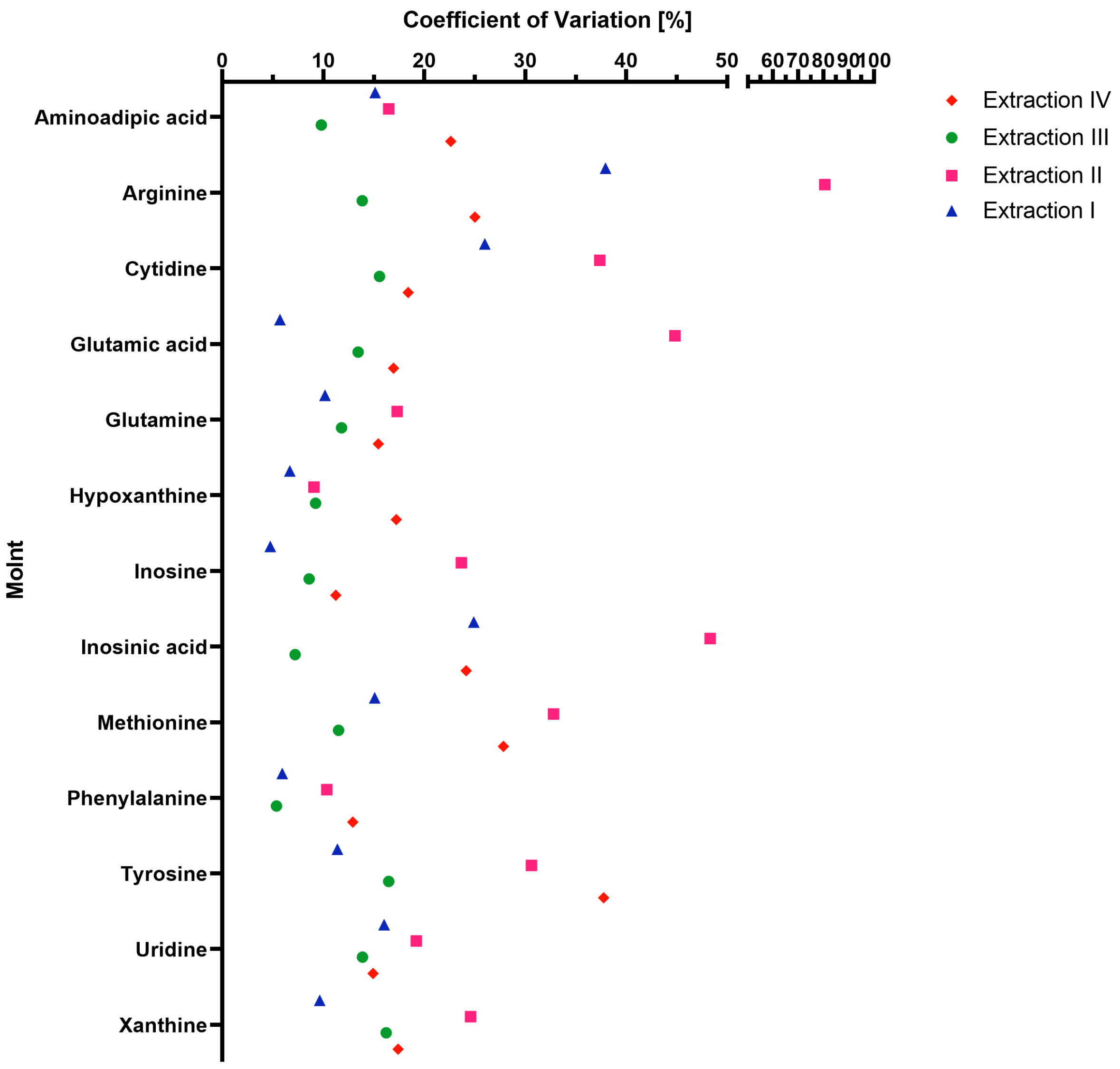

Figure S17. Coefficient of variation of each molecule of interest after analysis using the HILIC column and negative ionization. n= 5 for each sample group.

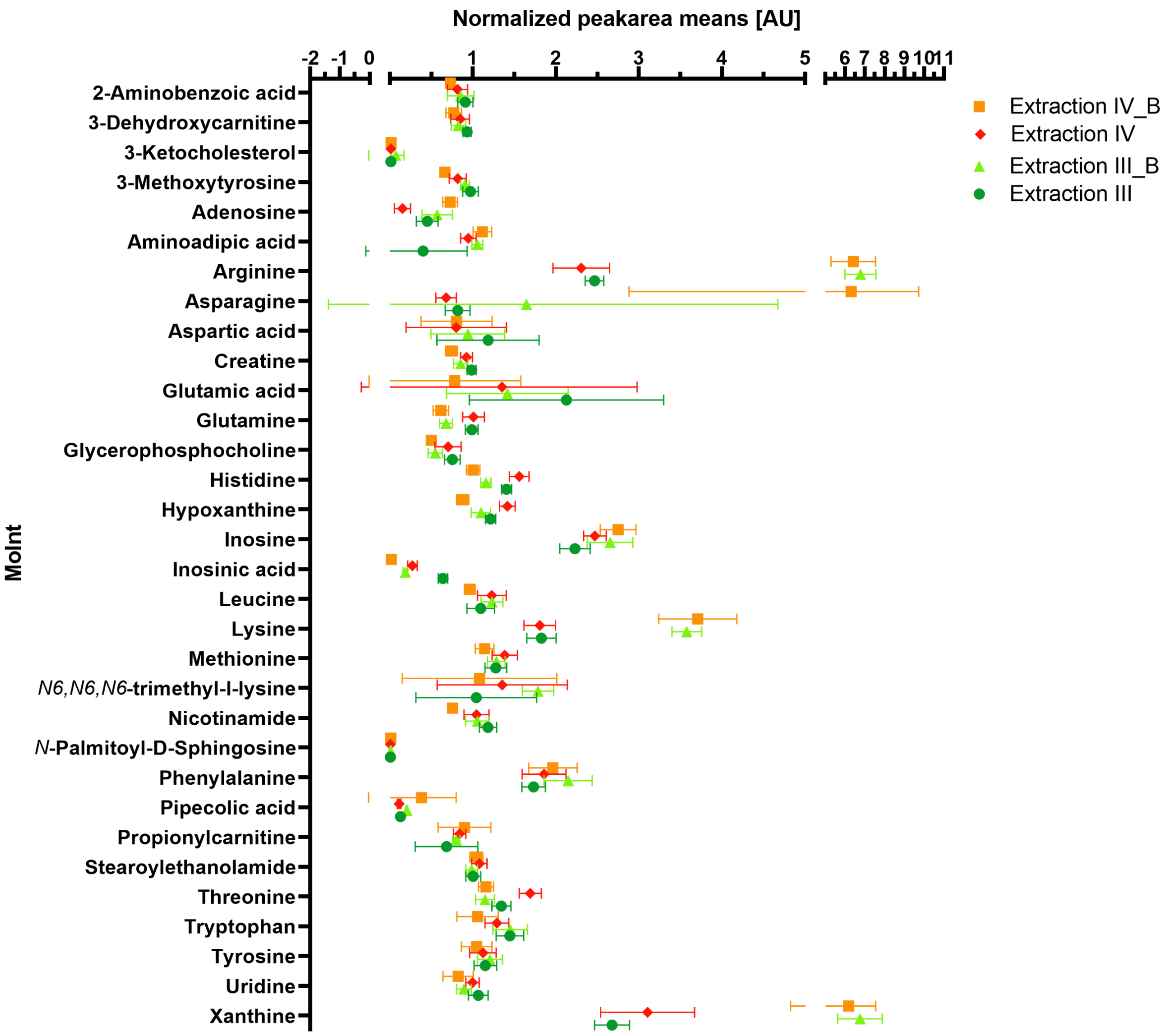

Figure S18. Normalized mean peak areas and standard deviation of each molecule of interest after analysis using the Phenyl-Hexyl column and positive ionization. n= 5 for each sample group. Addition of bead homogenization was denoted as “_B”.

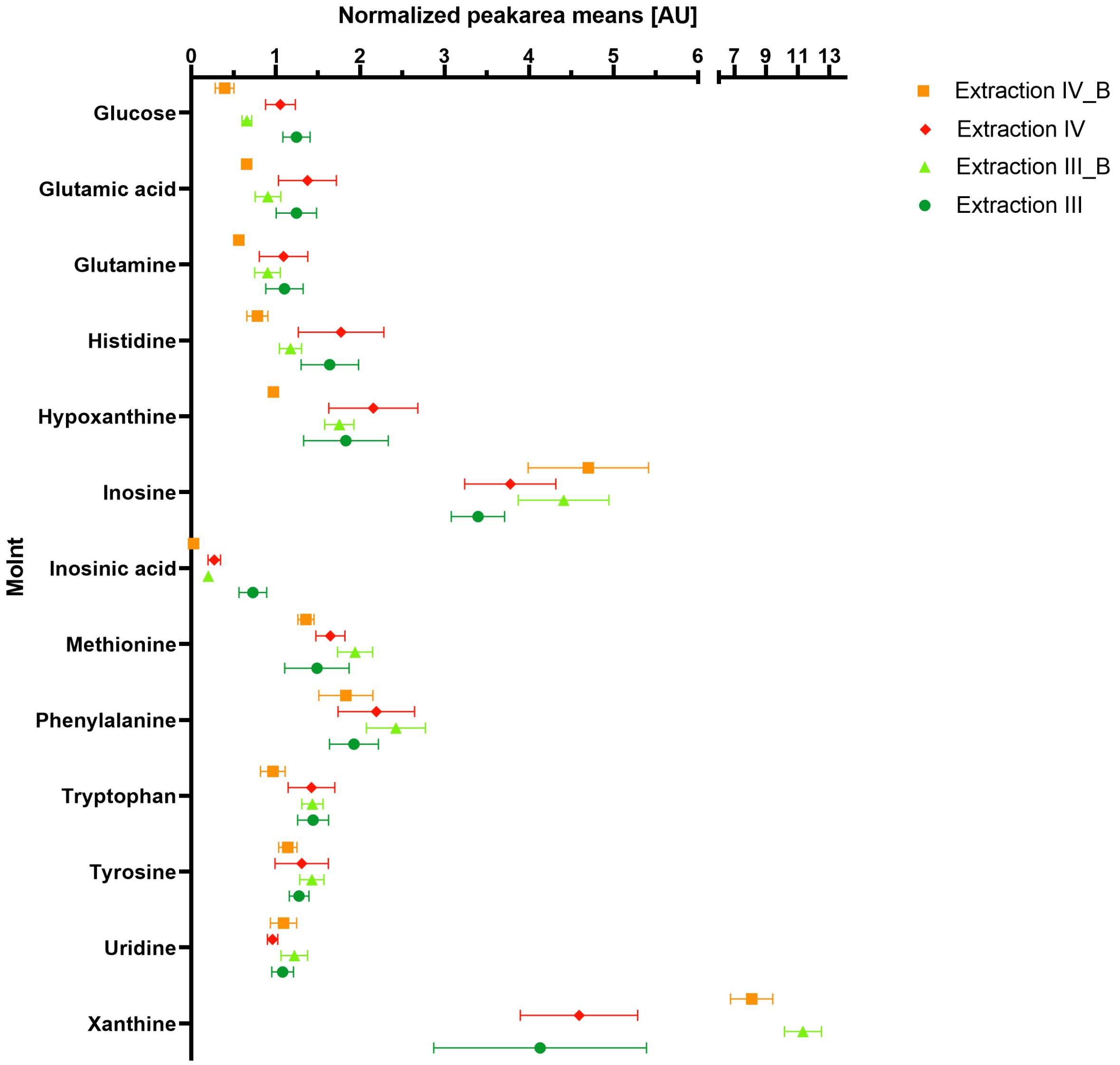

Figure S19. Normalized mean peak areas and standard deviation of each molecule of interest after analysis using the Phenyl-Hexyl column and negative ionization. n= 5 for each sample group. Addition of bead homogenization was denoted as “_B”.

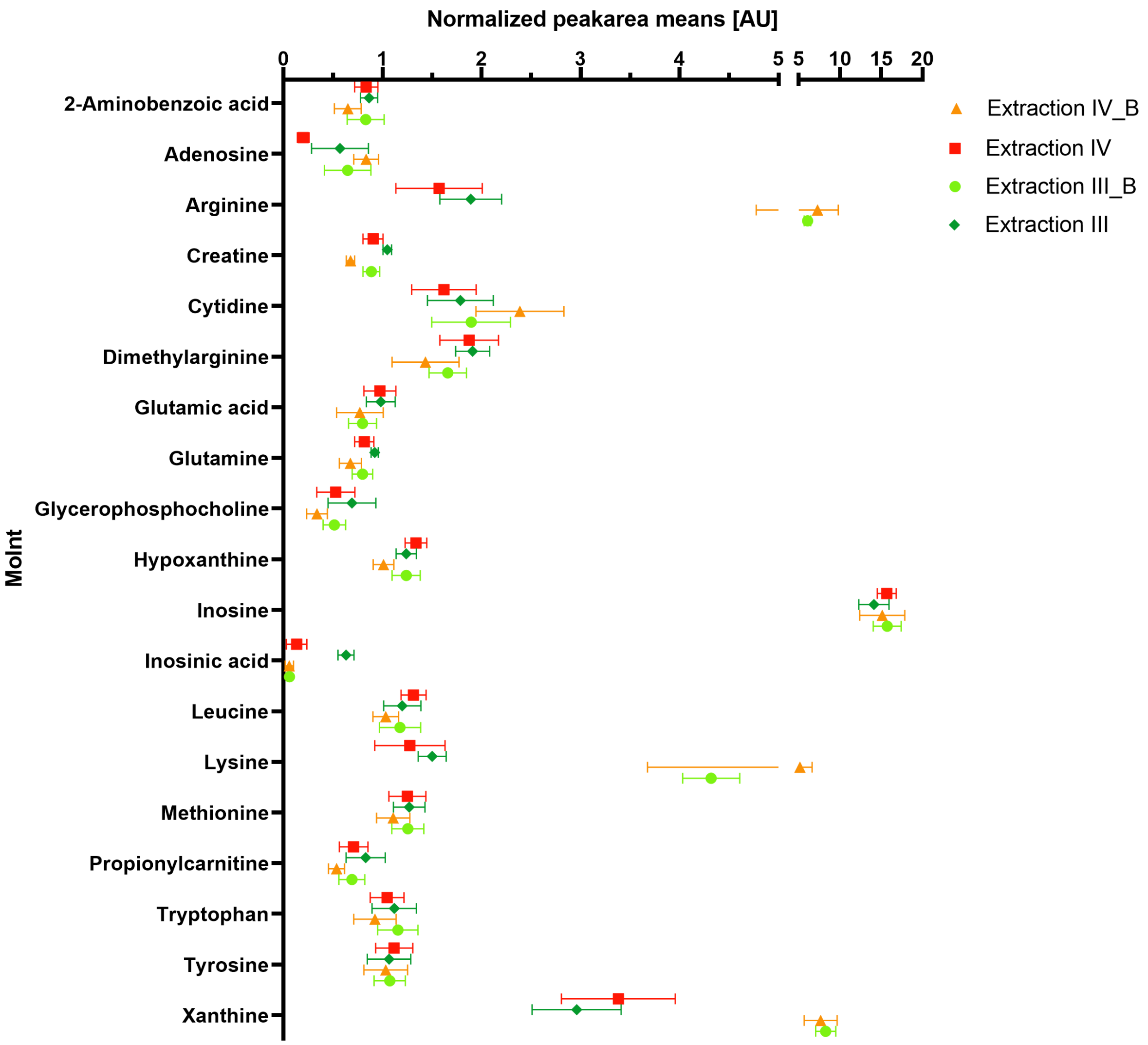

Figure S20. Normalized mean peak areas and standard deviation of each molecule of interest after analysis using the HILIC column and positive ionization. n= 5 for each sample group. Addition of bead homogenization was denoted as "_B".

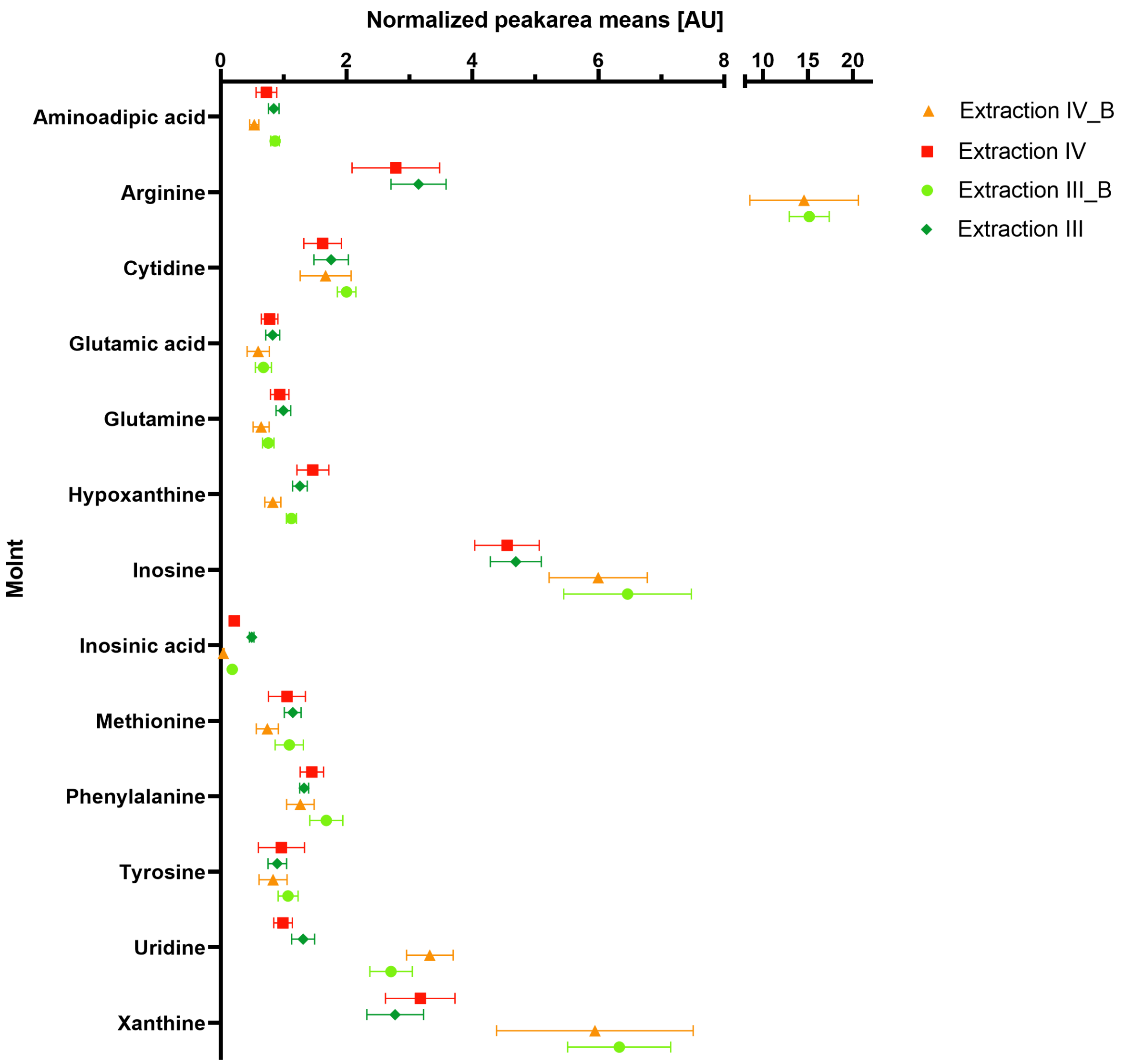

Figure S21. Normalized mean peak areas and standard deviation of each molecule of interest after analysis using the HILIC column and negative ionization. n= 5 for each sample group. Addition of bead homogenization was denoted as "_B".

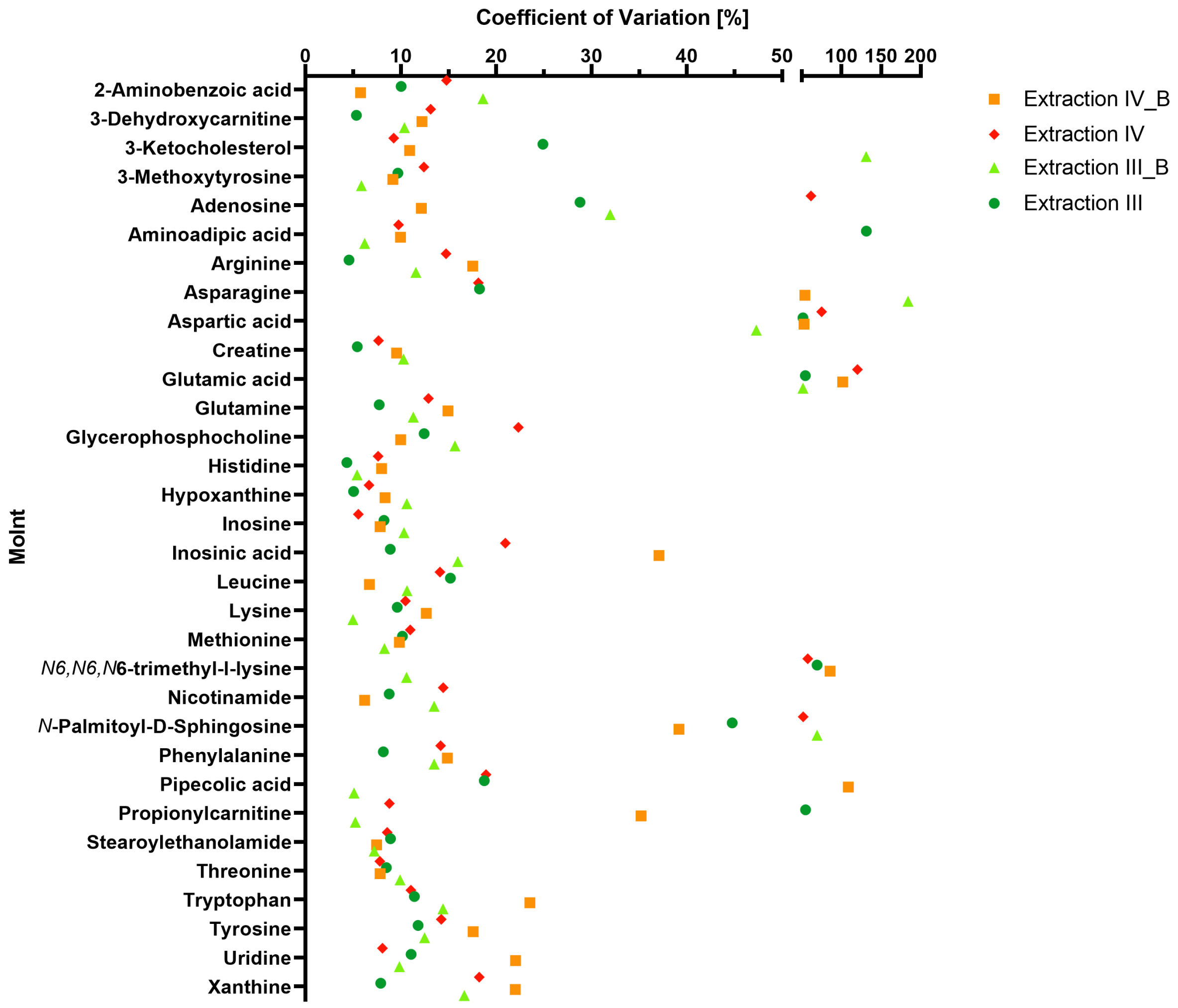

Figure S22. Coefficient of variation of each molecule of interest after analysis using the Phenyl-Hexyl column and positive ionization. n= 5 for each sample group. Addition of bead homogenization was denoted as "_B".

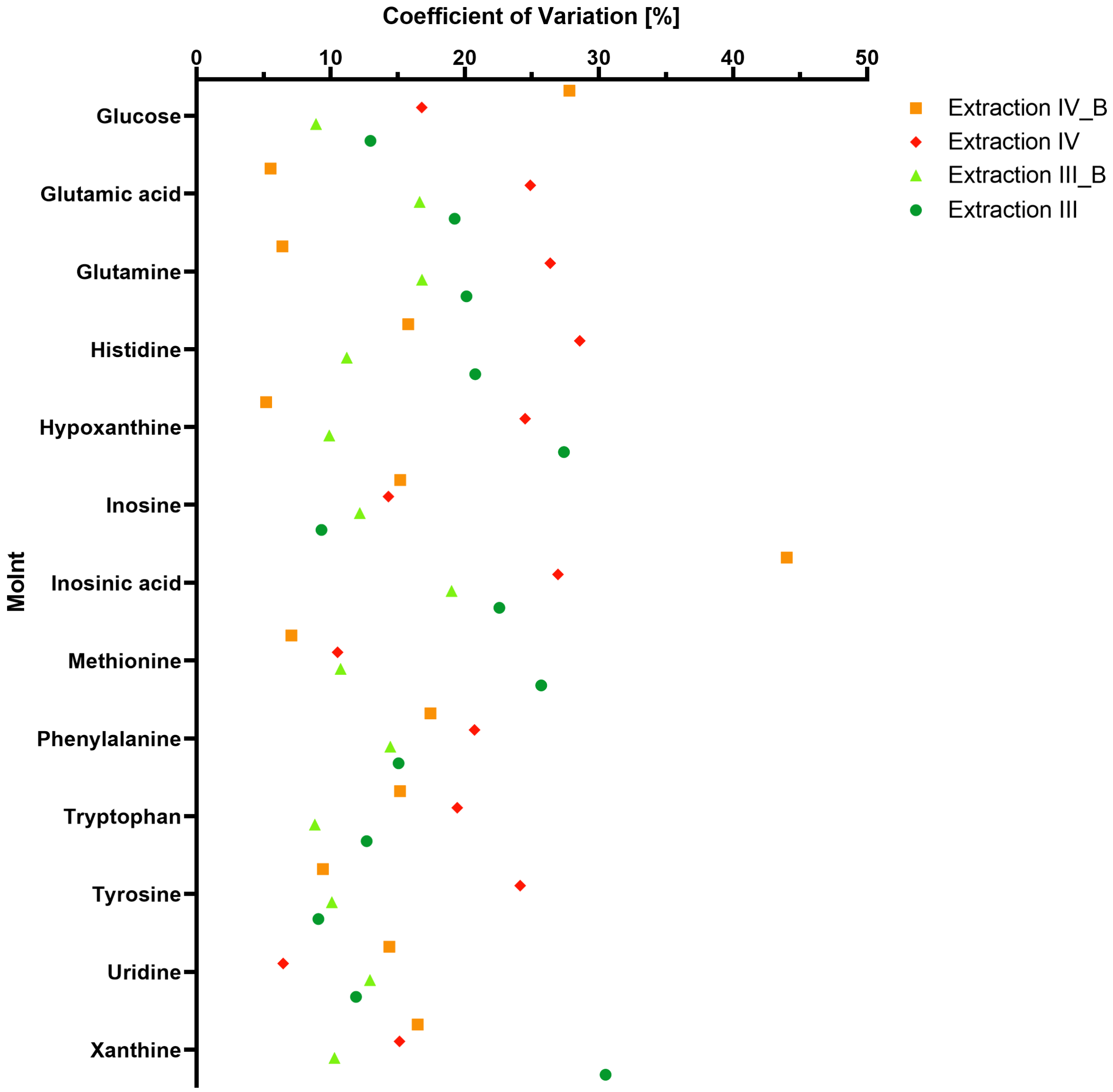

Figure S23. Coefficient of variation of each molecule of interest after analysis using the Phenyl-Hexyl column and negative ionization. n= 5 for each sample group. Addition of bead homogenization was denoted as "_B".

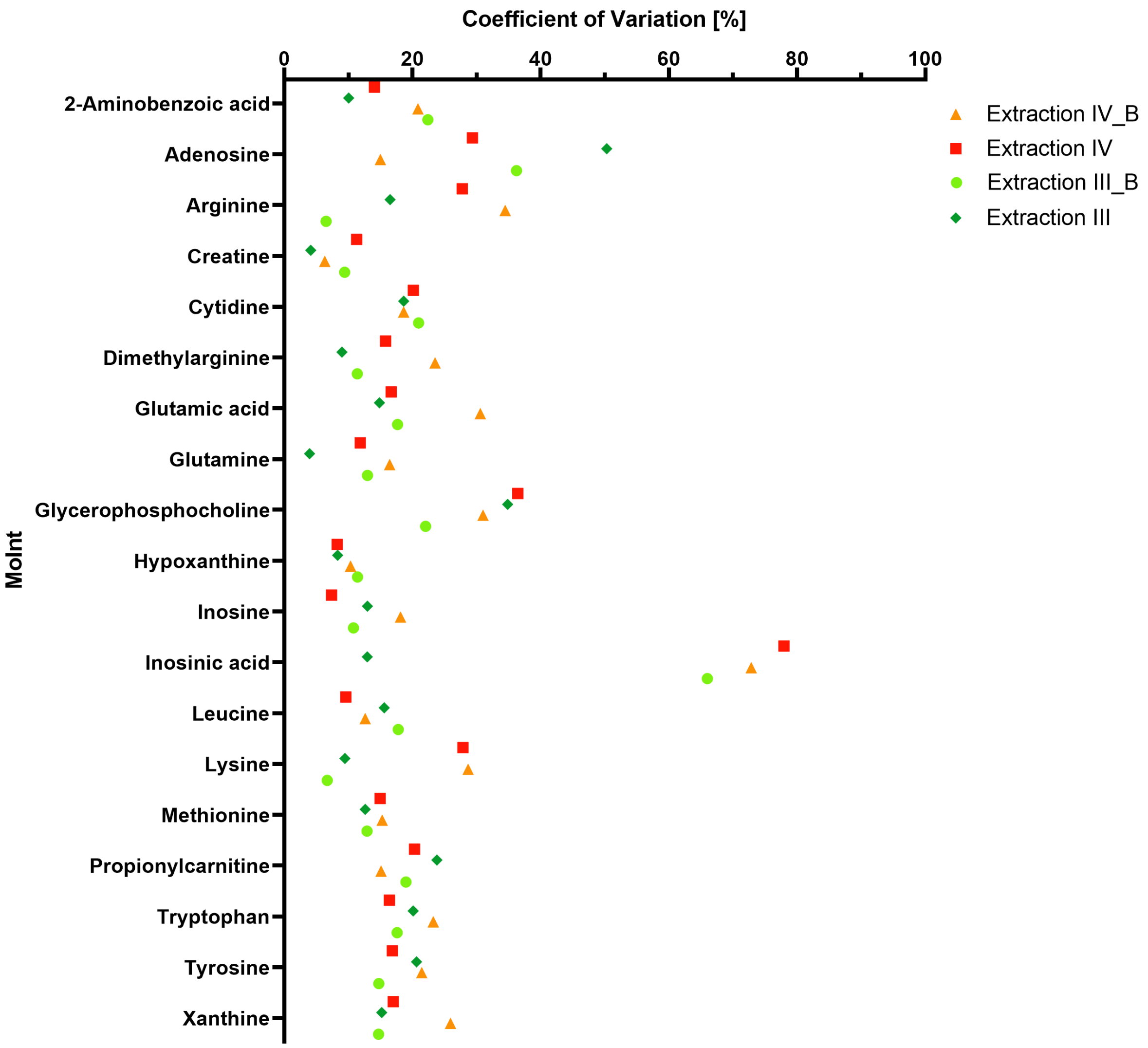

Figure S24. Coefficient of variation of each molecule of interest after analysis using the HILIC column and positive ionization. n= 5 for each sample group. Addition of bead homogenization was denoted as “_B”.

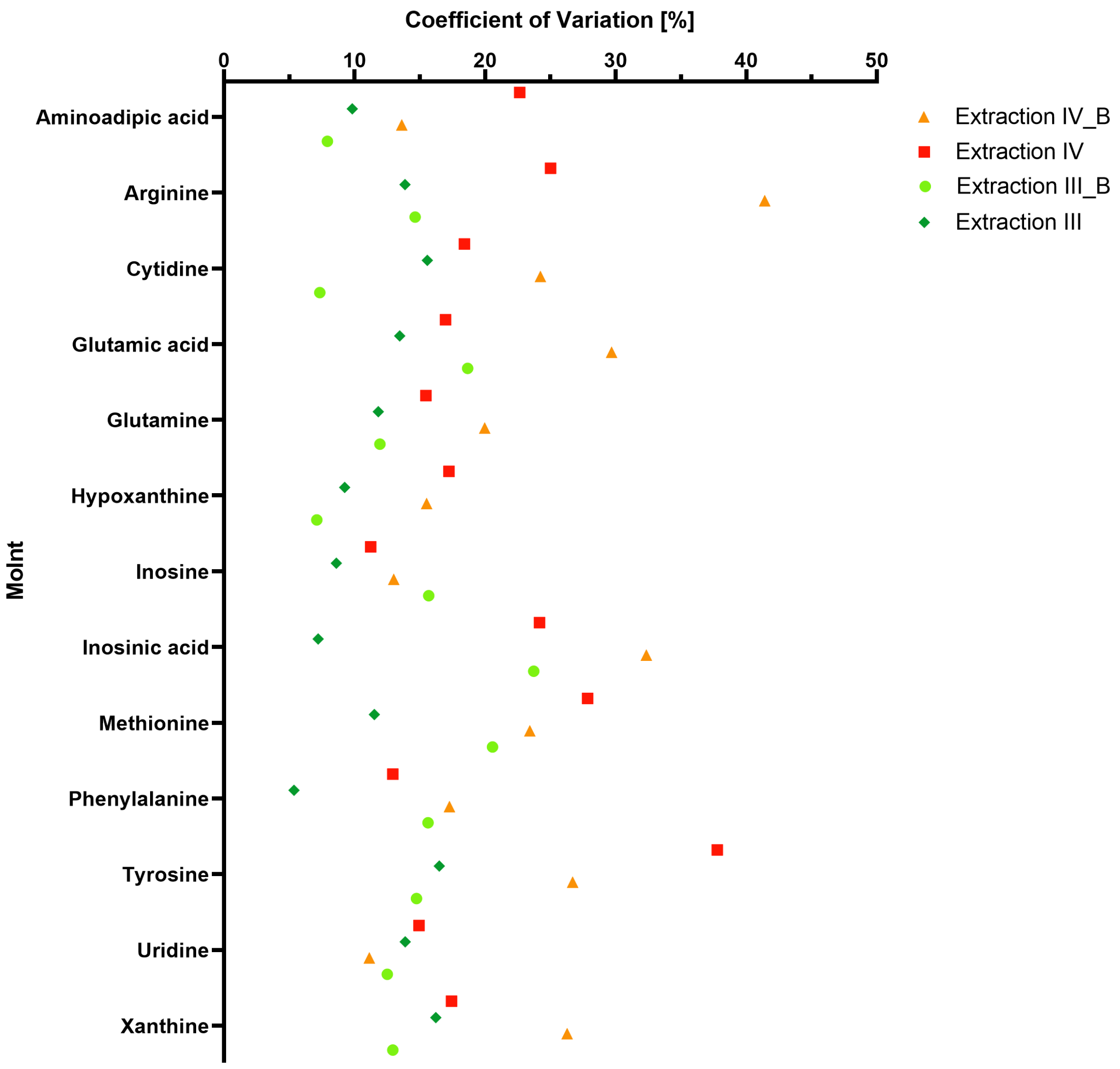

Figure S25. Coefficient of variation of each molecule of interest after analysis using the HILIC column and negative ionization. n= 5 for each sample group. Addition of bead homogenization was denoted as "_B".

References

Albors-Vaquer, A., A. Rizvi, M. Matzapetakis, P. Lamosa, A. V. Coelho, A. B. Patel, S. C. Mande, S. Gaddam, A. Pineda-Lucena, S. Banerjee and L. Puchades-Carrasco (2020). "Active and prospective latent tuberculosis are associated with different metabolomic profiles: clinical potential for the identification of rapid and non-invasive biomarkers." Emerg Microbes Infect **9**(1): 1131-1139.

Chai, T., F. Cui, Z. Yin, Y. Yang, J. Qiu and C. Wang (2016). "Chiral PCB 91 and 149 Toxicity Testing in Embryo and Larvae (Danio rerio): Application of Targeted Metabolomics via UPLC-MS/MS." Sci Rep **6**: 33481.

Conde, R., R. Laires, L. G. Gonçalves, A. Rizvi, C. Barroso, M. Villar, R. Macedo, M. J. Simões, S. Gaddam, P. Lamosa, L. Puchades-Carrasco, A. Pineda-Lucena, A. B. Patel, S. C. Mande, S. Banerjee, M. Matzapetakis and A. V. Coelho (2021). "Discovery of serum biomarkers for diagnosis of tuberculosis by NMR metabolomics including cross-validation with a second cohort." Biomed J.

Ding, Y., R. J. Raterink, R. Marin-Juez, W. J. Veneman, K. Egbers, S. van den Eeden, M. C. Haks, S. A. Joosten, T. H. M. Ottenhoff, A. C. Harms, A. Alia, T. Hankemeier and H. P. Spaink (2020). "Tuberculosis causes highly conserved metabolic changes in human patients, mycobacteria-infected mice and zebrafish larvae." Sci Rep **10**(1): 11635.

Park, Y. M., M. R. Meyer, R. Muller and J. Herrmann (2020). "Drug Administration Routes Impact the Metabolism of a Synthetic Cannabinoid in the Zebrafish Larvae Model." Molecules **25**(19).

Vrieling, F., B. Alisjahbana, E. Sahiratmadja, R. van Crevel, A. C. Harms, T. Hankemeier, T. H. M. Ottenhoff and S. A. Joosten (2019). "Plasma metabolomics in tuberculosis patients with and without concurrent type 2 diabetes at diagnosis and during antibiotic treatment." Sci Rep **9**(1): 18669.

Westerfield, M. (2007). The Zebrafish Book a Guide for the Laboratory Use of Zebrafish Danio* (Brachydanio) Rerio. Eugene, OR, University of Oregon Press.
